# Glycine promotes cardiomyocyte proliferation and heart regeneration via the GCN2/AKT signaling axis

**DOI:** 10.7150/thno.127992

**Published:** 2026-04-23

**Authors:** Feng Wang, Xia Zhang, Yang Tang, Yanji He, Wei Wang, Andy Zeng, Lijia Liang, Jing Wang, Chunyu Zeng

**Affiliations:** 1 Department of Cardiology, Daping Hospital, The Third Military Medical University, Chongqing, P. R. China.; 2 Key Laboratory of Geriatric Cardiovascular and Cerebrovascular Disease Research, Ministry of Education of China, Chongqing, P. R. China.; 3 Chongqing Key Laboratory for Hypertension Research, Cardiovascular Clinical Research Center, Chongqing Institute of Cardiology, Chongqing, P. R. China.; 4 Chongqing Institute of Green and Intelligent Technology, Chinese Academy of Sciences, Chongqing, P.R. China.; 5 State Key Laboratory of Trauma, Burns and Combined Injury, Daping Hospital, The Third Military Medical University, Chongqing, P. R. China.; 6 Department of Cardiology, The First Affiliated Hospital of Kunming Medical University, Kunming, P. R. China.; 7 Johns Hopkins University, Baltimore, MD 21205, USA.

**Keywords:** cardiomyocyte proliferation, amino acid metabolism, PI3K-AKT, GCN2, liposomal nanoformulation

## Abstract

**Rationale:**

While glucose-lipid metabolic remodeling is recognized as a major contributor to cardiomyocytes proliferation, the role of amino acid metabolism in cardiac regeneration remains poorly understood. In this study, glycine was identified as a previously unrecognized pro-proliferative amino acid.

**Methods:**

Primary cardiomyocytes, neonatal mice, and adult mouse models of myocardial infarction were employed to investigate the pro-proliferative effects of glycine on cardiomyocytes. Cardiomyocyte proliferation was assessed by immunofluorescence staining of cell-cycle-related markers. Cardiac function was evaluated by echocardiography, and histopathological alterations were examined using Masson's trichrome staining. To overcome the pharmacokinetic limitations of free glycine and enhance therapeutic efficacy, a cardiac-targeted liposomal nanoformulation (LNP@Gly) was developed and applied *in vivo*. Mechanistic studies were performed using RNA sequencing, Western blotting, co-immunoprecipitation (co-IP), immunostaining, pharmacological inhibition and activation approaches.

**Results:**

Exogenous glycine supplementation (700 mg/kg) consistently enhanced cardiomyocyte proliferation across *in vitro*, neonatal, and adult myocardial infarction models. In infarcted adult hearts, glycine supplementation further improved cardiac function and reduced myocardial fibrosis. A cardiac-targeted liposomal formulation of glycine (LNP@Gly) effectively overcame the pharmacokinetic limitations of free glycine and achieved enhanced therapeutic efficacy at a substantially lower dose (4 mg/kg). At the molecular level, glycine treatment was associated with increased AKT phosphorylation, reduced GCN2 activation, and weakened GCN2-AKT interaction. Pharmacological modulation further supported a critical role for the GCN2-AKT axis in glycine-induced cardiomyocyte proliferation and cardiac repair.

**Conclusions:**

These findings identify glycine as a novel metabolic regulator of cardiomyocyte proliferation and cardiac regeneration through modulation of the GCN2-AKT signaling axis. Moreover, the integration of amino acid-specific metabolic regulation with cardiac-targeted nanodelivery represents a translatable therapeutic strategy for ischemic heart disease.

## Introduction

Cardiovascular diseases, particularly myocardial infarction (MI), remain a leading cause of morbidity and mortality 1, responsible for millions of deaths annually and imposing a substantial burden on healthcare systems worldwide 2. A hallmark pathological feature of MI is the extensive, irreversible loss of cardiomyocytes, which constitutes the primary driver of adverse cardiac remodeling and subsequent progression to heart failure 3. Although current therapeutic strategies, including pharmacological interventions, catheter-based procedures, and surgical reperfusion, have significantly improved acute management and short-term survival, they don't mitigate the fundamental problem of cardiomyocyte loss or restore functional myocardium 4. Consequently, stimulating endogenous cardiomyocyte regeneration has emerged as a critical goal in cardiac repair and regenerative therapy.

Adult mammalian hearts, including those of humans, exhibit a limited intrinsic regenerative capacity, which is insufficient to compensate for the extensive cellular damage following ischemic injury 5, 6. In contrast, embryonic cardiomyocytes display robust proliferative potential that declines sharply after birth and becomes minimal within the first postnatal week 7. This postnatal decline in proliferative capacity coincides with marked changes in the cardiac environment and a well-characterized metabolic transition in cardiomyocytes. During embryogenesis, cardiac energy metabolism relies primarily on glycolysis, whereas the postnatal rise in oxygen tension drives a shift toward mitochondrial oxidative metabolism, with fatty acid β-oxidation becoming the predominant energy source 8-10. Accumulating evidence suggests that this metabolic switch contributes to cardiomyocyte cell cycle exit and regenerative failure 11. Notably, interventions targeting glucose and lipid metabolism regulators have been shown to restore cardiomyocyte proliferation and improve cardiac repair 12-16. Beyond glucose and lipid metabolism, amino acid metabolism also undergoes dynamic remodeling during the perinatal period 9, 17. However, its role in cardiomyocyte proliferation remains poorly defined. Although recent studies have implicated specific amino acid derivatives in modulating cell cycle activity 18, a systematic understanding of amino acid metabolism in myocardial regeneration is still lacking.

In this study, we identify glycine as a previously unrecognized pro-proliferative metabolic regulator that enhances cardiomyocyte proliferation and promotes cardiac regeneration following ischemic injury. Mechanistically, glycine-mediated regeneration is governed by the GCN2-AKT signaling axis, and its therapeutic potential is achieved with improved efficiency by a cardiac-targeted liposomal nanoformulation (LNP@Gly). Our findings provide new insights into the underlying amino acid metabolic regulation of heart regeneration, and propose a translationally viable therapeutic strategy for ischemic cardiomyopathy via precision-targeted metabolic intervention.

## Methods and materials

### Isolation of neonatal rat ventricular myocytes (NRVMs) and drug administration

Neonatal rat cardiomyocytes were isolated using a modified density gradient centrifugation method 19. Briefly, hearts were harvested from 1-2-day-old and 7-day-old Sprague-Dawley rats and immersed in cold ADS buffer to remove blood. Ventricular tissues were minced into 1 mm^3^ fragments and subjected to serial enzymatic digestion using 300U/mL collagenase II (Worthington, LS004176) at 37 ℃ with gentle agitation. The supernatant containing dissociated cells was collected after each digestion cycle and pooled. The pooled cell suspension was centrifugation at 300 g for 5 minutes to pellet the cells, which were then resuspended in ADS buffer. To enrich cardiomyocytes, a discontinuous percoll (Cytiva, 17-0891-01) gradient was prepared, and the cell suspension was layered on top. Following centrifugation at 1800 g for 30 minutes without brake, cardiomyocytes were collected from the interface between the high- and low-density layers. The collected cardiomyocytes were washed twice with ADS and resuspended in DMEM medium (Zeta Life, BM0003) supplemented with 10% fetal bovine serum (Gibco, 10082147) and 1% penicillin-streptomycin (Beyotime, C0222). Cardiomyocytes were plated onto gelatin-coated dishes or glass coverslips and incubated at 37 °C with 5% CO₂. After 24 hours, non-adherent cells were removed by medium replacement, and cardiomyocytes were subsequently subjected to different treatments.

Amino acids were directly dissolved in culture medium to achieve final concentrations of 2, 5, 10, and 20 mM (threonine, T8441, cysteine, C7352, glycine, G8790, proline, P5607, serine, S4311, Sigma-Aldrich). The PI3K-AKT inhibitor LY294002 (MCE, 154447-36-6) was prepared as a stock solution in DMSO and subsequently diluted in culture medium to a working concentration of 20 μM. Similarly, the GCN2 activator halofuginone (Caymen Chem, 13370) was dissolved in DMSO to generate a stock solution and then diluted in culture medium to a final concentration of 20 nM for experimental use.

### Immunofluorescence staining

Immunostaining of paraffin-embedded tissue sections and cultured primary cardiomyocytes (CMs) was performed as previously described^14^. For cultured primary CMs, cells were fixed with 4% paraformaldehyde for 15 minutes at room temperature, permeabilized with 0.2% Triton X-100, and blocked with 5% bovine serum albumin (BSA) for 1 hour. Cells were then incubated overnight at 4 °C with primary antibodies against cardiac troponin T (CTNT, Invitrogen, MA5-12960, 1:200), Ki67 (CST, 9027, 1:200), phospho-histone H3 (CST, 3377, 1:200), GCN2 (Santa Cruz, sc-374609, 1:200), and AKT (CST, 4691S, 1:200). For co-localization analysis, cells were co-incubated with anti-GCN2 and anti-AKT antibodies, followed by species-specific fluorophore-conjugated secondary antibodies for 1 hour at room temperature. Nuclei were counterstained with DAPI, and images were captured using a confocal laser scanning microscope (CLSM, Olympus FluoView 1200, Japan).

For paraffin-embedded heart tissue sections, 5 μm-thick sections were deparaffinized, rehydrated, and subjected to antigen retrieval in citrate buffer (pH 6.0). After blocking with 5% BSA, sections were incubated with primary antibodies against CTNT (Invitrogen, MA5-12960, 1:200), Ki67 (CST, 9027, 1:200) and pH3 (CST, 3377, 1:200) overnight at 4 °C, followed by secondary antibody incubation and DAPI counterstaining. Fluorescence images were acquired with a fluorescence microscope.

### Wheat germ agglutinin (WGA) staining

WGA staining of paraffin-embedded heart tissue sections was performed as previously described 20. Briefly, 5 μm-thick heart sections were deparaffinized, rehydrated, and subjected to heat-induced antigen retrieval. Following cooling to room temperature and through washing with PBS, sections were incubated with Alexa Fluor-conjugated wheat germ agglutinin (WGA, Invitrogen, W11261) for 30 minutes at room temperature in the dark. Nuclei were counterstained with DAPI, followed by washing with PBS. Fluorescence images were acquired using a fluorescence microscope. Cardiomyocyte membrane boundaries were visualized, and cell cross-sectional area was quantified using ImageJ software.

### Western blot and Co-IP

Heart tissues were homogenized in RIPA lysis buffer containing protease inhibitor cocktail (MCE, HY-K0012), and total protein was extracted by centrifugation at 4℃. Protein concentrations were determined using a BCA protein assay kit (Beyotime, P0012). Equal amounts of protein (30-50 μg) were separated by SDS-PAGE and transferred onto nitrocellulose filter NC membranes. Membranes were blocked with 5% BSA for 1 hour at room temperature and incubated overnight at 4°C with primary antibodies against AKT (CST, 4691S, 1:1000; Proteintech, 10176-2-ap, 1:1000), phospho-AKT (CST, 4060S, 1:1000), GCN2 (CST, 3302S, 1:1000), phospho-GCN2 (BIOSS, BS-3155R, 1:1000), and GAPDH (Solarbio, M1000110, 1:5000). After washing, membranes were incubated with species-specific fluorescent secondary antibodies (Li-Cor, IRDye 800 CW, goat anti-rabbit,1:10,000) for 1 hour at room temperature in the dark. Fluorescent signals were detected using the Odyssey Imaging System, and band intensities were quantified using ImageJ or Image Studio software.

For co-ip, magnetic agarose beads (Thermo Fisher, A36797) were first incubated with anti-AKT (CST, 4691S) or anti-GCN2 (BIOSS, BS-3155R) antibodies at 4 °C for 2 hours with gentle rotation to allow antibody-bead binding. After washing to remove unbound antibodies, the beads were then incubated with 500-1000 μg of total protein extracted from heart tissues overnight at 4 °C. Following extensive washing with IP buffer to remove nonspecific binding, the immunoprecipitated protein complexes were eluted by boiling in SDS sample buffer, separated by SDS-PAGE, and analyzed by fluorescent Western blotting to assess the interaction between AKT and GCN2.

### Animal experiments

All procedures were conducted in accordance with the NIH Guide for the Care and Use of Laboratory Animal and were approved by the Laboratory Animal Welfare and Ethics Committee of the Army Medical University (Approval No. AMUWEC20247148). Neonatal Sprague-Dawley rats at postnatal day 1 (P1) and day 7 (P7) were used for the isolation of primary cardiomyocytes. For *in vivo* evaluation of the postnatal proliferative window, C57BL/6J mice at P7 received intraperitoneal injections of glycine or vehicle control and were analyzed at designated time points. Adult C57BL/6J mice (8-10 weeks old) were used to establish the MI model via permanent ligation of the left anterior descending coronary artery, followed by corresponding treatment protocols. P1 and P7 rats were briefly anesthetized by hypothermia on ice for 3-4 minutes to induce deep anesthesia, after which the hearts were rapidly excised for cell isolation. For MI surgery and echocardiographic analysis, mice were anesthetized with 3% isoflurane in an induction chamber and maintained under 1.5-2% isoflurane (delivered in 100% oxygen) via a nose cone. At the designated experimental endpoints, mice were humanely euthanized by cervical dislocation under deep anesthesia with 3% isoflurane.

### Myocardial infarction model

MI was induced in adult C57BL/6J mice (8-10 weeks old) using a rapid, non-ventilated surgical protocol, as previously described^21^. Briefly, mice were anesthetized with isoflurane (3% for induction and 1.5-2% for maintenance, delivered in 100% oxygen via inhalation) and placed supine on a heated surgical pad. A left thoracotomy (~5 mm incision) was made between the fourth and fifth ribs to expose the heart without endotracheal intubation or mechanical ventilation. The left anterior descending (LAD) coronary artery was identified and ligated with 7-0 silk suture within 1 minute of incision. The chest wall was immediately closed, and anesthesia discontinued to allow spontaneous breathing. Sham-operated mice underwent the same procedure without LAD ligation.

### Mosaic analysis with double markers (MADM) mice

To more accurately assess the proliferation of cardiomyocytes, we employed the Mosaic Analysis with Double Markers (MADM) system. This approach was implemented by crossing three transgenic mouse lines obtained from the Jackson Laboratory: Igs2tm2^(ACTB-tdTomato-EGFP)Zng^/J (cat. no. 022977), Igs2tm2^(ACTB-EGFP- tdTomato)Zng^/J (cat. no. 022976), and FVB (129)-A1cf ^Tg (Myh6-cre/Esr1)1Jmk^/J (cat. no. 005657) to generate Myh6^mERcremER^-MAD. To induce genetic recombination, tamoxifen (sigma, T5648) was administered via intraperitoneal injection at a dose of 10 mg/kg daily for three consecutive days, starting one day after MI surgery. Cardiac tissues were harvested two weeks post-MI, and labeled cardiomyocytes were identified by immunofluorescence detection of GFP and RFP.

### Quantification of glycine level

Glycine levels in mouse heart tissue and plasma were quantified using the glycine assay kit (Fluorometric) (Abcam, ab211100) according to the manufacturer's protocol. For plasma collection, blood was obtained via retro-orbital puncture using heparin-coated capillary tubes to prevent coagulation. Plasma was isolated by centrifugation at 2000 g for 20 min at 4 °C. For cardiac tissue analysis, 20 mg of heart tissue was homogenized in glycine assay buffer on ice and centrifuged at 13,000 g for 10 min to remove debris.

### RNA-seq and data analysis

Primary cardiomyocytes were treated with or without glycine for 24 hours, and total RNA was extracted using a standard TRIzol protocol according to the manufacturer's instructions (Invitrogen, 15596026). RNA purity and concentration were assessed using a NanoDrop spectrophotometer and Agilent Bioanalyzer. RNA-seq libraries were prepared and sequenced using the Illumina platform to generate paired-end reads. Sequencing data were deposited in the NCBI Gene Expression Omnibus (GEO) database under GSE318305.

Transcriptomic data were analyzed using NCBI online RNA-seq analysis toolkit, which includes quality control, read alignment, and differential expression analysis. Gene expression was normalized and expressed as fragments per kilobase of transcript per million mapped reads (FPKM). Differentially expressed genes (DEGs) between glycine-treated and control groups were identified based on fold change and adjusted p-values. Functional enrichment analysis, including Gene Ontology (GO) and Kyoto Encyclopedia of Genes and Genomes (KEGG) pathway analysis, was performed using integrated NCBI tools.

### Echocardiographic evaluation

Transthoracic echocardiography was performed on mice at 0, 3, 14, 28 days after MI surgery using the Vevo 3100 high-resolution ultrasound system. Mice were anesthetized with 2 % isoflurane and maintained on a heated platform to preserve body temperature. Parasternal long-axis and short-axis M-mode images were acquired at the level of the papillary muscles. At least three cardiac cycles were recorded per view. Left ventricular ejection fraction (EF%), left ventricular fractional shortening (FS%), left ventricular internal diastolic diameter (LVIDd) and left ventricular internal systolic diameter (LVIDs) were derived from left ventricle dimensions in the 2D long-axis view by using Vevo LAB software.

### Measurement of cardiac fibrosis

Cardiac fibrosis was assessed at 4 weeks after myocardial infarction. Hearts were harvested, fixed in 4% paraformaldehyde, embedded in paraffin, and sectioned transversely from apex to base into 5 μm thick short-axis slices at multiple levels. Sections were stained using a Masson's trichrome staining kit (Solarbio, G1340) according to the manufacturer's instructions. In the stained sections, collagen-rich fibrotic tissue stained blue, whereas viable myocardium appeared red.

Infarct size was quantified by angular measurement on short-axis sections using ImageJ software. For each section, the infarct arc (the angle spanning the fibrotic region along the endocardial border) and the total left ventricular circumference (360°) were measured. The infarct size was expressed as a percentage of the total LV circumference using the following formula: Infarct size (%) = (infarct arc angle / 360) × 100%. Multiple levels were analyzed for each heart, and the mean infarct size per heart was calculated. All image analysis was performed in a blinded fashion.

### Synthesis of cardiac target peptide (DSPE-PEG2K-CSTSMLKAC)

100 mg of DSPE-PEG2K-NHS dissolved in 3 ml of DMF. Subsequently, CSTSMLKAC peptide (110 mg) and triethylamine (300 mg) were added, and the mixture was stirred until complete dissolution was achieved. The reaction was carried out at room temperature for 12 hours. After reaction, the product was dialyzed with a molecular weight cutoff of 2 000 Da to remove unreacted peptide and PEG moieties. The dialyzed solution was collected, lyophilized, and analyzed by ^1^H nuclear magnetic resonance (NMR, Bruker Avance III 400 Spectrometer).

### Preparation and characterization of the LNP@Gly delivery system

The lipid nanoformulation was prepared using the film dispersion method 22. Briefly, 0.1 g of the lipid formulation components, including soy phosphatidylcholine (SPC), cholesterol and cardiac-targeting peptide (CSTSMLKAC) modified DSPE-PEG were precisely weighed and then dissolved in 1 ml of trichloromethane in a round bottom flask. The organic solvent was subsequently evaporated under vacuum conditions to form a homogeneous thin lipid film. For drug loading, 15 mg of glycine was dissolved in 12 mL of deionized water and then added to the flask containing the dried lipid film. Then, the system underwent controlled hydration until complete film dispersion was visually confirmed. Finally, the resulting liposome suspension was subjected to sonication and extruded through a 100-nm membrane (Whatman) using an Avanti mini extruder to obtain glycine-loaded cardiotropic lipid nanoparticles (LNP@Gly). As a control, the blank nanocarrier (LNP) were prepared using the same procedures but with deionized water (without glycine) was used during the hydration step.

The average hydrodynamic diameter and zeta potential of the lipid nanoparticles were measured using Dynamic Light scattering (DLS, a Zetasizer Nano ZSP (Malvern Instruments Ltd., Malvern, UK). The morphology of LNP@Gly and LNP was characterized by Transmission Electron Microscopy (TEM, JEOL, Japan) operated at a voltage of 200 keV. For TEM sample preparation, droplets of LNP@Gly and LNP suspensions were deposited onto 200-mesh carboncoated copper grids (Zhongjingkeyi Technology, China), followed by staining with 1% phosphotungstic acid and subsequent drying prior to TEM analysis.

For the stability study, nanoparticle samples were stored in saline and plasma, respectively. Particle size was measured periodically by dynamic light scattering (DLS) over the course of 11 days. Glycine entrapment efficiency (EE) and drug loading (DL) were determined using the glycine assay kit (Abcam, ab211100). For liposomal nanoparticle formulations, 0.3% Triton X-100 was added to the supernatant to disrupt lipid membranes and fully release encapsulated glycine. Prior to analysis, all samples were filtered through 10 kDa molecular weight cutoff ultrafiltration tube. EE was calculated as follows: % EE = (amount of encapsulated glycine/ initial amount of glycine) × 100. DL was calculated as follows: % DL = (amount of encapsulated glycine/ total amount of LNP@Gly) × 100.

### Safety evaluation for LNP@Gly

To test the biosafety of LNP@Gly* in vivo*, the male C57BL/6J mice were randomly divided into three groups and administered saline, LNP or LNP@Gly (6.25 mg/kg) via tail vein injection every 2 days, respectively. After 2 weeks, all mice were sacrificed, and the blood along with major organs (heart, liver, spleen, lung, and kidneys) were collected for biochemical analysis and H&E staining. The body weights of the mice were recorded every two days throughout the experimental period.

### *Ex vivo* biodistribution and cardiac targeting efficiency of LNP@Gly

The MI mouse model was established as previously described. Following MI induction, equivalent doses of Cy5.5-labeled glycine and LNP@Gly (both containing 4 mg/kg glycine) were administered via tail vein injection. At 2 and 24 hours post-injection, heart and major organs were harvested for *ex vivo* fluorescence quantification using an *in vivo* imaging system (IVIS Lumina XR, Caliper, USA). To assess the cardiomyocyte-targeting specificity, hearts were harvested and immediately embedded in optimal cutting temperature (OCT) Compound (Fisher HealthCare) and cryosectioned into 8-μm thick sections using a cryostat Leica CM 1950 (Leica BioSystems, Nussloch). Immunofluorescence staining was performed using mouse antibodies to CTNT (Invitrogen, MA5-12960, 1:200) and Alexa Fluor™ 488-conjugated secondary antibody (Invitrogen, A-11008, 1:200). The nuclei were stained with DAPI. Finally, fluorescence images of heart were acquired by confocal microscopy with consistent acquisition parameters across all samples.

### *Ex vivo* therapeutic efficacy of LNP@Gly in myocardial infarction mice

C57BL/6 J mice were used to establish MI mouse model. Following confirmation of successful MI induction, mice were randomly into four experimental groups and treated with glycine, LNP, LNP@Gly (with a dosage of 4 mg/kg of glycine) or saline, respectively. All treatment regimens were administered via tail-vein injection every two days for a duration of two weeks.

### Statistical analysis

All statistical analyses were performed using GraphPad Prism version 8.0. Data are presented as mean ± standard error of the mean (SEM). Comparisons between two groups were assessed using a two-tailed unpaired Student's t-test. For comparisons among more than two groups, one-way analysis of variance (ANOVA) followed by Dunnett's or Tukey's post hoc test was applied as appropriate. For experiments involving two independent variables, two-way ANOVA with either the Bonferroni or Sidak's multiple-comparison test was used. A value of *P* < 0.05 was considered statistically significant.

## Results

### Identification of glycine as a pro-proliferative amino acid in cardiomyocytes

To explore the role of amino acid metabolism in regulating cardiomyocyte proliferation, we analyzed metabolomic datasets from cardiac tissues of postnatal day 1 (P1) and day 7 (P7) mice 17. It revealed a marked metabolic reprogramming of amino acid profiles during the early postnatal period. In particular, the myocardial levels of five amino acids, including threonine, cysteine, glycine, proline and serine, were significantly lower in P7 hearts compared with P1 hearts (Figure S1A), coinciding with the sharp decline in intrinsic cardiac regenerative capacity during the first postnatal week.

**Figure 1:**
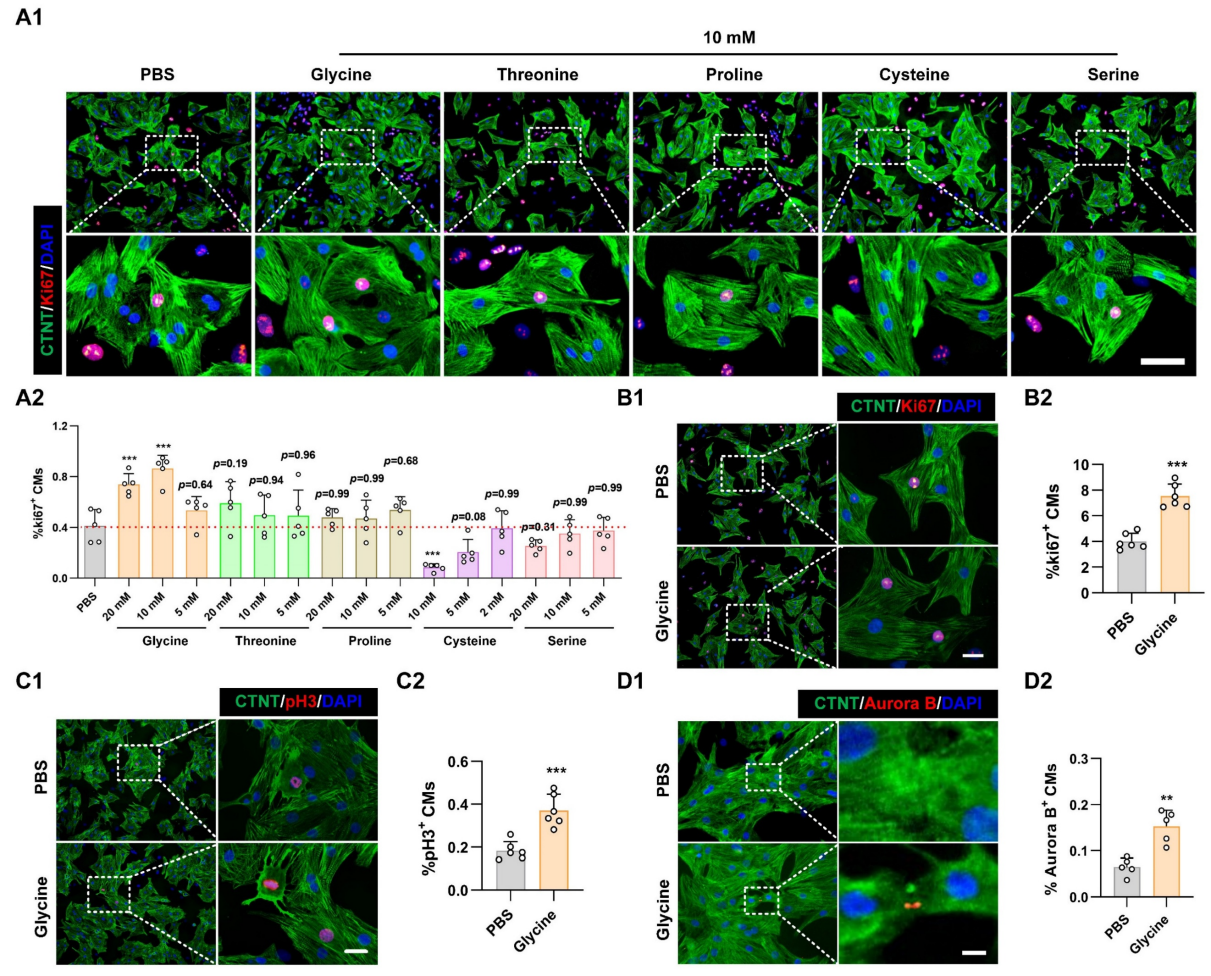
** Glycine promotes cardiomyocyte proliferation. (A)** Representative immunofluorescence images (A1) and corresponding quantification (A2) of Ki67^+^ cardiomyocytes in P7 cardiomyocytes treated with threonine, cysteine, glycine, proline, or serine (2, 5, 10, or 20 mM) for 24 hours. Scale bar = 40 μm. **(B-D)** Representative immunofluorescence images (B1-D1) and corresponding quantification (B2-D2) of Ki67⁺, pH3⁺, and Aurora B⁺ cardiomyocytes in P1 cardiomyocytes treated with 10 mM glycine for 24 hours. Scale bar = 20 μm (Ki67 and pH3) and 5 μm (Aurora B). Statistical analysis was performed using one-way ANOVA followed by Tukey's multiple-comparison test (A2) and two-tailed unpaired Student's *t*-test (B2-D2) (*n* = 5, **P < 0.01, ***P < 0.001, vs. PBS).

To determine whether supplementation with these amino acids, whose levels were lower in P7 cardiomyocytes, could rescue cardiomyocyte proliferative potential, primary cardiomyocytes isolated from P7 rat hearts were cultured and treated with varying concentrations (2 mM, 5 mM, 10 mM, and 20 mM) of candidate amino acids. Remarkably, Ki67 immunostaining, a canonical proliferation marker, revealed that among all the tested amino acids, only glycine significantly increased the proportion of Ki67-positive cardiomyocytes (Figure 1A and Figure S1B). We next assessed whether the pro-proliferative effect of glycine was restricted to P7 cardiomyocytes by testing its impact on primary cardiomyocytes isolated from P1 hearts. Consistent with the P7 findings, glycine treatment significantly increased the proportion of Ki67, phosphorylated histone H3 (pH3) and Aurora B kinase (Aurora B)-positive cardiomyocytes (Figure 1B-D). Collectively, these findings identify glycine as a key pro-proliferative amino acid in primary cardiomyocytes and highlight its potential role in modulating cardiac regenerative capacity.

**Figure 2:**
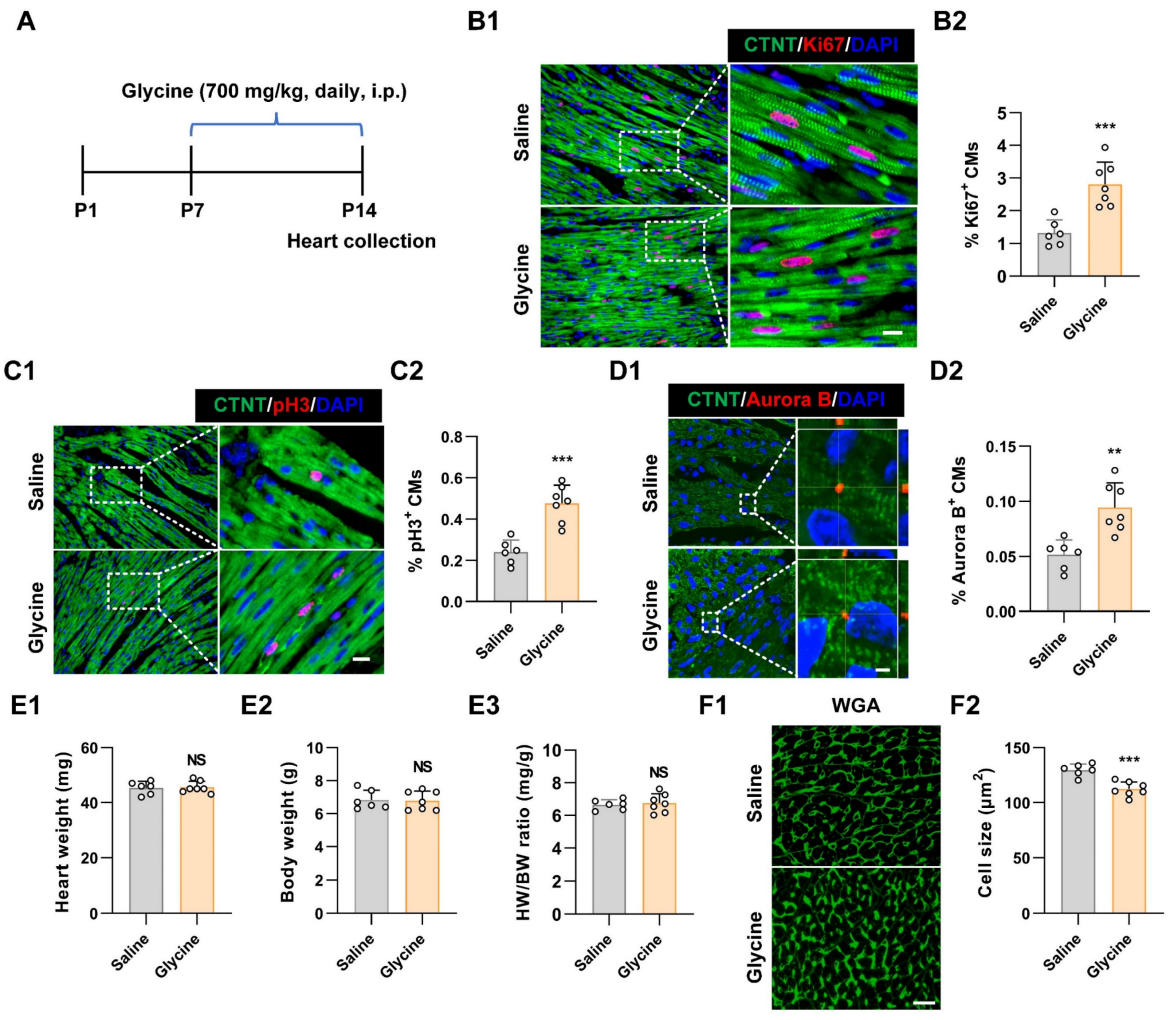
** Glycine supplementation extends the postnatal cardiac regenerative window. (A**) Schematic diagram of the *in-vivo* experimental design. P7 mice received daily intraperitoneal injections of glycine (700 mg/kg/day) for 7 consecutive days, and hearts were harvested at P14. i.p., intraperitoneal. **(B-D)** Representative immunofluorescence images (B1-D1) and quantification (B2-D2) of Ki67^+^, pH3^+^, Aurora B^+^ cardiomyocytes in saline-treated (control) and glycine-treated hearts (700 mg/kg/day, i.p.). Scale bar = 10 μm (Ki67 and pH3) and 2 μm (Aurora B).** (E)** Quantification of heart weight (E1), body weight (E2), and heart-to-body weight ratio (E3) at P14 of saline-treated and glycine-treated mice (700 mg/kg/day, i.p.). **(F)** Representative image (F1) and quantification (F2) of WGA staining in P14 hearts after 1 week of daily intraperitoneal injection of either saline or glycine (700 mg/kg/day, i.p.). Scale bar = 20 μm. Data are presented as mean ± SEM. Statistical significance was determined using a two-tailed unpaired Student's *t*-test (*n* = 6-7, NS = Not significant, **P < 0.01, ***P < 0.001 vs. saline).

To further assess whether glycine promotes cardiomyocyte proliferation *in vivo*, we administered glycine daily via intraperitoneal injection to postnatal P7 mice for seven consecutive days. Hearts were harvested at P14 for proliferation and histological analyses (Figure 2A). Based on previously published studies 23, 24, we evaluated multiple glycine doses (500, 700, and 900 mg/kg) in this postnatal developmental model. Among the tested doses, 700 mg/kg exhibited the most pronounced pro-proliferative effect as demonstrated by immunofluorescence staining for Ki67, pH3 and Aurora B (Figure 2B-D and Figure S2A-C). Although cardiomyocyte proliferation was significantly enhanced, no significant difference was observed in the heart weight-to-body weight (HW/BW) ratio between glycine-treated and control groups (Figure 2E), indicating that glycine supplementation did not induce cardiac hypertrophy or gross structural alterations. This finding aligns with previous research demonstrating that glycine administration prevents pressure-overload-induced cardiac hypertrophy 23. Importantly, cardiomyocyte cross-sectional area in glycine-treated hearts was smaller than that in saline group, determined by wheat germ agglutinin (WGA) staining (Figure 2F), implying an increase in cardiomyocyte number in glycine-treated hearts, which was consistent with the immunofluorescence results. Together, these results demonstrate that glycine supplementation effectively reactivates cardiomyocyte proliferation and extends the postnatal cardiac regenerative capacity.

### Glycine promoted adult cardiac regeneration and repair after MI

**Figure 3:**
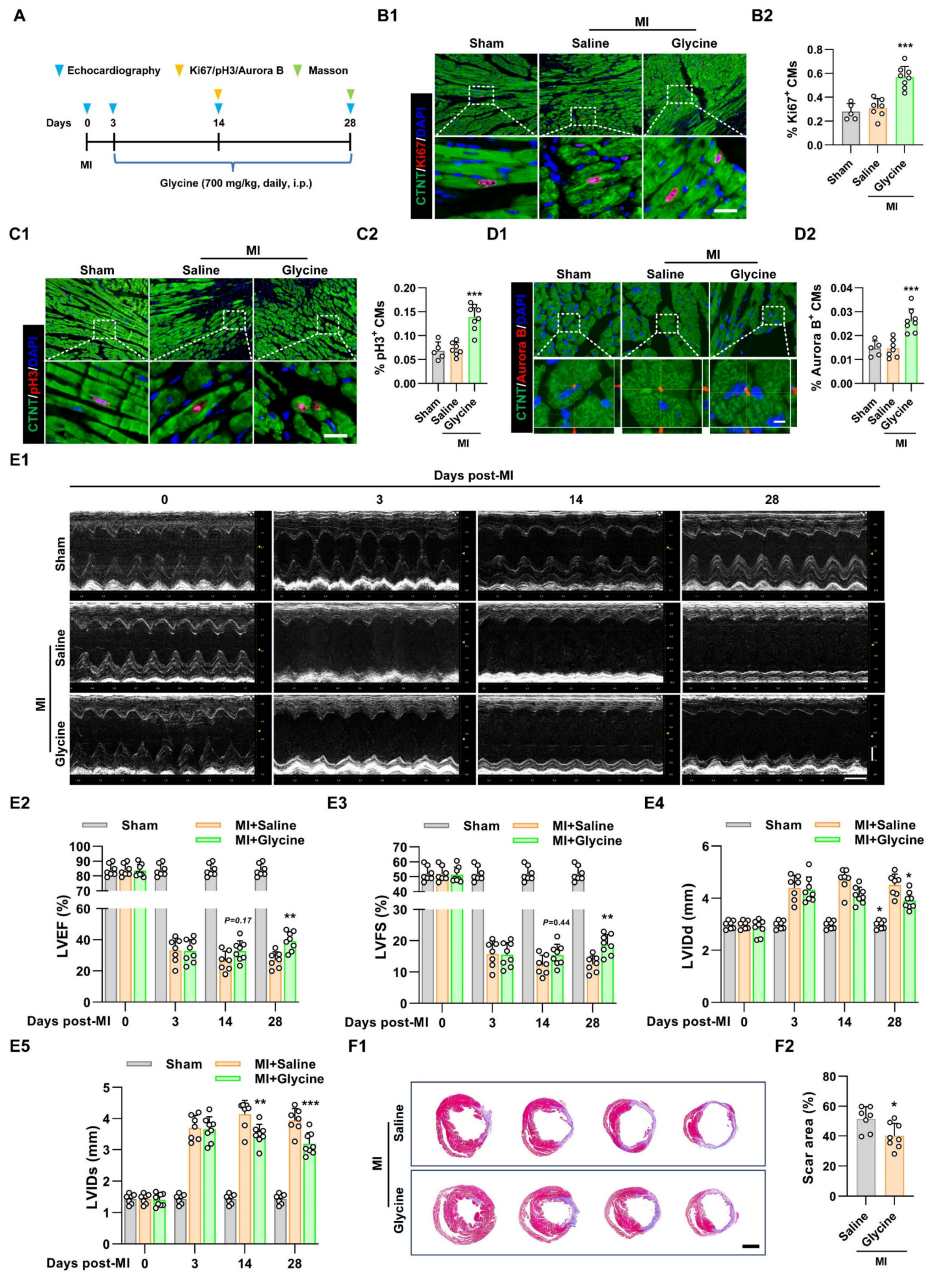
** Glycine promotes adult cardiac regeneration and repair after myocardial infarction. (A)** Schematic diagram illustrating the administration of glycine and the experimental design in the MI mouse model. i.p., intraperitoneal. MI, myocardial infarction. **(B-D)** Representative immunofluorescence images (B1-D1) and corresponding quantification (B2-D2) of Ki67⁺, pH3⁺, and Aurora B⁺ cardiomyocytes in hearts 14 days post-MI, with or without glycine treatment (700 mg/kg/day, i.p.). Scale bar = 20 μm (Ki67 and pH3) and 5 μm (Aurora B).** (E)** Representative echocardiographic images (E1) and quantification of left ventricular ejection fraction (LVEF %, E2), fraction shortening (LVFS %, E3), left ventricular internal diastolic diameter (LVIDd, E4) and left ventricular internal systolic diameter (LVIDs, E5) at 0, 3, 14, and 28 days post-MI. **(F)** Representative images (F1) and quantification (F2) of Masson's trichrome staining of hearts 28 days post-MI, Scale bar = 1.5 mm. Data are shown as mean ± SEM. Statistical analysis was performed using one-way ANOVA followed by Tukey's multiple-comparison test (B2, C2 and D2), two-way ANOVA with Sidak's post-hoc multiple-comparison test (E2-E5) and two-tailed unpaired Student's *t*-test (F2) (sham group, *n* = 5; MI group, *n* = 7-8; *P < 0.05, **P < 0.01, ***P < 0.001 vs. saline).

To systematically evaluate the therapeutic potential of glycine supplementation in promoting post-ischemic cardiac regeneration and functional recovery, we established an adult murine model of MI by permanent ligation of the left anterior descending (LAD) coronary artery. Pharmacological intervention with daily intraperitoneal glycine administration was initiated on day 3 post-MI following confirmatory echocardiographic verification of ischemic injury (Figure 3A). Using a dose-comparison approach in the MI model, we also tested multiple glycine doses (500, 700, and 900 mg/kg) and found that 700 mg/kg produced the most pronounced cardiomyocyte proliferative response, as indicated by increased Ki67^+^, pH3^+^, and Aurora B^+^ staining (Figure 3B-D and Figure S3A-C). Echocardiographic assessment further showed that glycine-treated mice exhibited improved cardiac function, as evidenced by increased left ventricular ejection fraction (LVEF) and left ventricular fractional shortening (LVFS) and decreased in left ventricular dilatation (Figure 3E). Moreover, Masson's trichrome staining performed at 28 days post-MI showed a marked reduction in infarct size in glycine-treated hearts (Figure 3F), indicating improved structural preservation and reduced myocardial damage. Importantly, glycine treatment did not induce detectable changes in cardiac structure and function in sham-operated mice (Figure S3D-E). Taken together, these results demonstrate that glycine supplementation promotes cardiomyocyte proliferation, attenuates infarct expansion, and significantly improves cardiac functional recovery following ischemic injury.

### Glycine stimulates cardiomyocyte proliferation and promotes cardiac repair via the PI3K-AKT pathway

**Figure 4:**
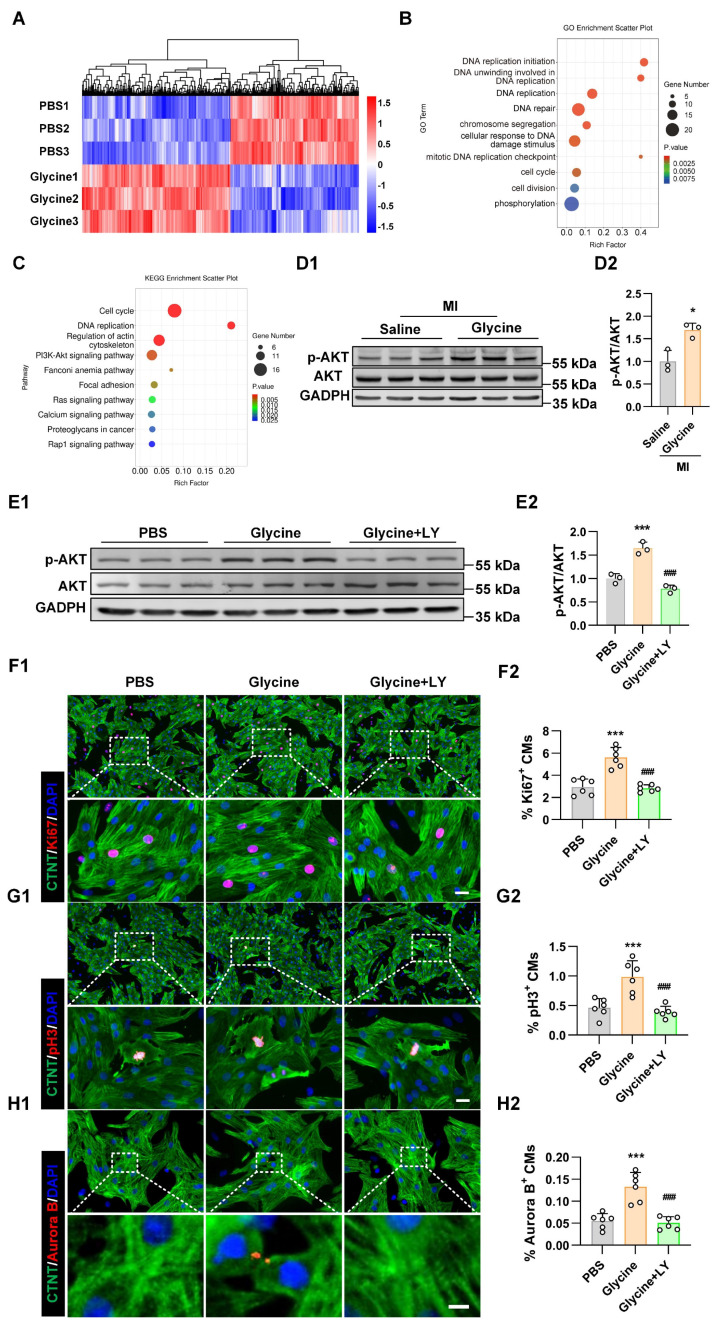
** Glycine activates PI3K-AKT signaling pathway in cardiomyocytes. (A)** Heatmap of differentially expressed genes identified by RNA sequencing in primary cardiomyocytes treated with glycine (10 mM, 24 hours). **(B)** Gene Ontology (GO) enrichment analysis of genes upregulated by glycine treatment. **(C)** Kyoto Encyclopedia of Genes and Genomes (KEGG) pathway enrichment analysis of genes upregulated by glycine treatment. **(D)** Representative Western blot images (D1) and quantification (D2) of phosphorylated AKT (p-AKT) levels in myocardial tissue 14 days post-MI, with glycine (700 mg/kg/day, i.p.) and saline treatment. Data are presented as mean ± SEM. Statistical significance was determined using a two-tailed unpaired Student's *t*-test (*n* = 3, *P < 0.05 vs. saline).** (E)** Representative Western blot images (E1) and quantification (E2) of phosphorylated AKT (p-AKT) levels in P1 cardiomyocytes treated with glycine (10 mM, 24 hours), PI3K inhibitor LY294002 (LY, 20 μM, 24 hours) and PBS (control) treatment. Data are presented as mean ± SEM. Statistical significance was determined using a one-way ANOVA followed by Tukey's multiple-comparison test (*n* = 3, *P < 0.05 vs. PBS). **(F-H)** Representative immunofluorescence images (F1-H1) and corresponding quantification (F2-H2) of Ki67⁺, pH3⁺, and Aurora B⁺ cardiomyocytes in P1 cardiomyocytes treated with glycine (10 mM, 24 hours) in the presence or absence of the PI3K inhibitor LY294002 (20 μM, 24 hours). LY, LY294002. Scale bar = 20 μm (Ki67 and pH3) and 5 μm (Aurora B). Data are presented as mean ± SEM. Statistical analysis was performed using one-way ANOVA followed by Tukey's multiple-comparison test (*n* = 6, ***P < 0.001 vs. PBS; ^###^*P* < 0.001 vs. glycine).

To elucidate the molecular mechanisms underlying the pro-proliferative effects of glycine on cardiomyocytes, we performed transcriptome analysis on primary cardiomyocytes treated with glycine. RNA-sequencing revealed substantial transcriptional changes, with 788 genes upregulated and 698 genes downregulated compared to untreated controls (Figure 4A). Gene Ontology (GO) enrichment analysis of the upregulated genes showed significant enrichment in biological processes related to DNA replication, cell cycle progression, and mitotic division (Figure 4B). Kyoto Encyclopedia of Genes and Genomes (KEGG) pathway analysis further revealed that PI3K-AKT signaling pathway was activated following glycine supplementation (Figure 4C). The PI3K-AKT signaling pathway has been demonstrated to play a pivotal role in driving cardiomyocyte cell cycle activity and facilitating myocardial regeneration^25^-^29^. To elucidate the role of the PI3K-AKT signaling pathway in glycine-induced cardiomyocyte regeneration, we examined the activity of AKT and found that its phosphorylation level was significantly increased in glycine-treated heart tissues (Figure 4D). To future determine whether the pro-proliferative effect of glycine depends on PI3K-AKT signaling, we performed pharmacological blockade experiments using the PI3K inhibitor LY294002 (20 μM, 24 hours). Western blot analysis confirmed that LY294002 effectively suppressed glycine-induced AKT phosphorylation, indicating efficient inhibition of PI3K-AKT signaling (Figure 4E). Immunofluorescence staining showed a significant reduction in the proportions of Ki67^+^, pH3^+^, and Aurora B^+^ cardiomyocytes following LY294002 co-treatment (Figure 4F-H). Collectively, these results demonstrate that glycine promotes cardiomyocyte proliferation *in vitro* through PI3K-AKT signaling.

**Figure 5:**
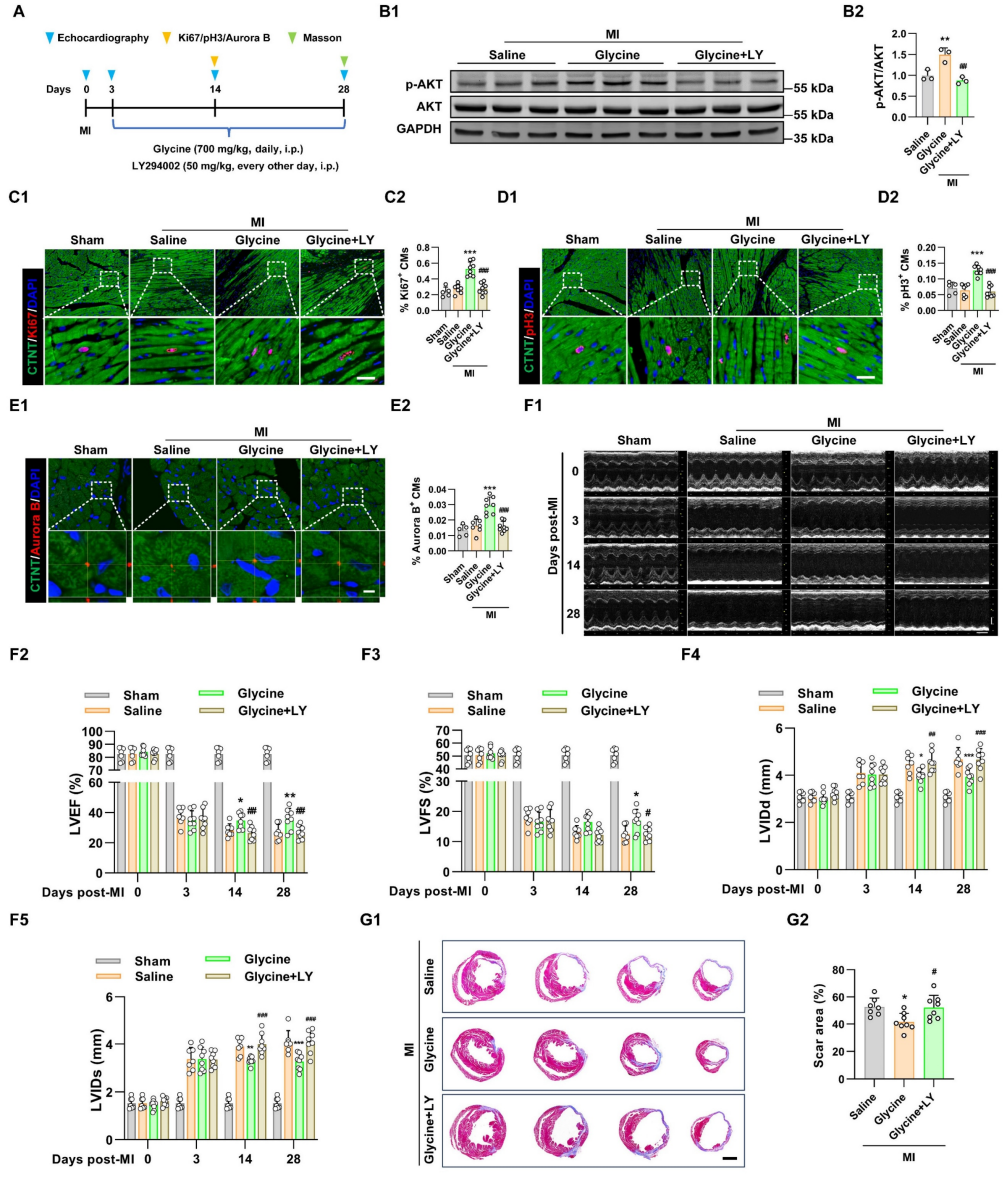
** Glycine promotes adult cardiac regeneration and repair through PI3K-AKT signaling. (A)** Schematic diagram of the experimental design illustrating PI3K-AKT pathway inhibition with LY294002 during glycine treatment in adult mice after MI. **(B)** Representative Western blot images (B1) and quantification (B2) of phosphorylated AKT (p-AKT) levels in myocardial tissue 14 days post-MI, with glycine (700 mg/kg/day, i.p.), LY (LY294002, 50 mg/kg, every other day, i.p.) and saline (control) treatment. Data are presented as mean ± SEM. Statistical analysis was performed using one-way ANOVA followed by Tukey's multiple-comparison test (*n* = 3, **P < 0.01 vs. saline; ^##^*P* < 0.01 vs. glycine). **(C-E)** Representative immunofluorescence images (C1-E1) and corresponding quantification (C2-E2) of Ki67⁺, pH3⁺, and Aurora B⁺ cardiomyocytes in hearts 14 days post-MI treated with glycine (700 mg/kg/day, i.p.), LY294002 (LY, 50 mg/kg, every other day, i.p.), or saline. Scale bar = 20 μm (Ki67 and pH3) and 5 μm (Aurora B). Data are presented as mean ± SEM. Statistical analysis was performed using a one-way ANOVA followed by Tukey's multiple-comparison test (sham group, *n* = 5; MI group, *n* = 7-8, ***P < 0.001 vs. saline; ^###^*P* < 0.001 vs. glycine). **(F)** Representative echocardiographic images (F1) and quantification of left ventricular ejection fraction (LVEF %, F2), fraction shortening (LVFS %, F3), left ventricular internal diastolic diameter (LVIDd, F4) and left ventricular internal systolic diameter (LVIDs, F5) at 0, 3, 14, and 28 days post-MI, with glycine (700 mg/kg/day, i.p.), LY (LY294002, 50 mg/kg, every other day, i.p.) and saline treatment. Data are shown as mean ± SEM. Differences among groups were analyzed by two-way ANOVA with Sidak's post-hoc multiple-comparison test (sham group, *n* = 5; MI group, *n* = 7-8, *P < 0.05, **P < 0.01, ***P < 0.001 vs. saline; ^#^*P* < 0.05, ^##^*P* < 0.01, ^###^*P* < 0.001 vs. glycine). **(G)** Representative images (G1) and quantification (G2) of Masson's trichrome staining of hearts at 28 days post-MI, with glycine (700 mg/kg/day, i.p.), LY (LY294002, 50 mg/kg, every other day, i.p.) and saline treatment. Scale bar = 1.5 mm. Data are presented as mean ± SEM. Statistical analysis was performed using one-way ANOVA followed by Tukey's multiple-comparison test (*n* = 7-8, *P < 0.05 vs. saline; ^#^*P* < 0.05 vs. glycine).

We next examined the role of PI3K-AKT pathway in glycine-mediated cardiac proliferation and repair *in vivo*. Adult mice subjected to MI were treated with glycine starting on day 3 post-MI. In parallel, a cohort of mice received concurrent administration of the PI3K inhibitor LY294002 (50 mg/kg, every other day, i.p.) to block PI3K-AKT signaling during glycine treatment (Figure 5A). Co-treatment with the PI3K inhibitor LY294002 effectively blocked glycine-induced AKT phosphorylation in myocardial tissue, as confirmed by Western blot analysis (Figure 5B). Functionally, inhibition of the PI3K-AKT pathway abolished the glycine-mediated stimulatory effect on cardiomyocyte proliferation, assessed by Ki67, pH3 and Aurora B staining (Figure 5C-E). Notably, echocardiographic analysis showed that glycine-treated mice failed to show observed improvement in cardiac function upon PI3K-AKT pathway inhibition (Figures 5F). Furthermore, histological evaluation at 28 days post-MI demonstrated glycine-mediated reduction in infarct size was disappeared by co-treatment with LY294002 (Figure 5G). Consequently, these findings demonstrate that glycine promotes adult cardiac regeneration and functional recovery after MI in a PI3K-AKT-dependent pathway.

**Figure 6:**
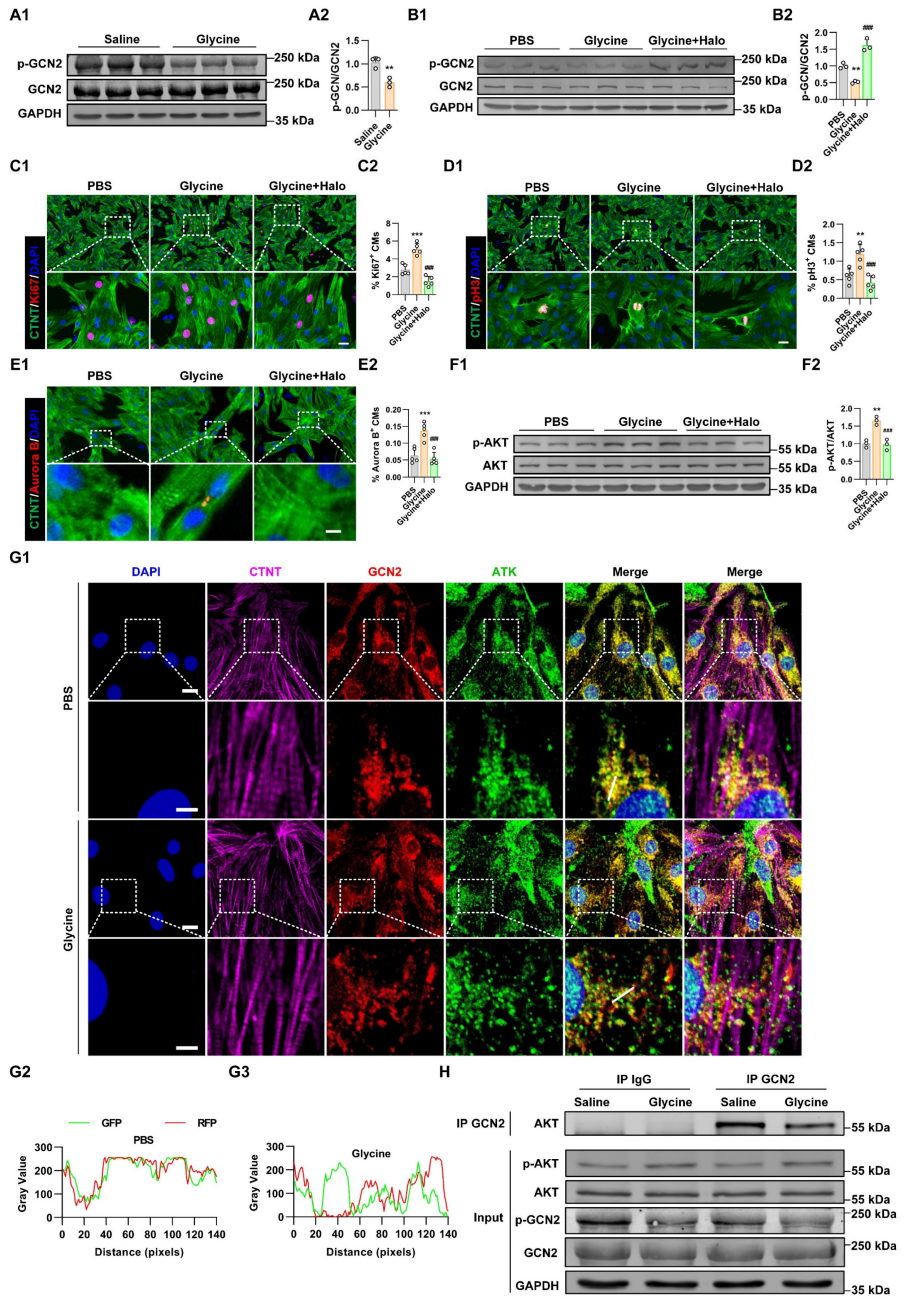
** GCN2 mediates glycine sensing and AKT activation in cardiomyocytes. (A)** Representative Western blot images (A1) and quantification (A2) of phosphorylated GCN2 (p-GCN2) levels in myocardial tissue 14 days post-MI, with glycine (700 mg/kg/day, i.p.) and saline (control) treatment. Data are presented as mean ± SEM. Statistical significance was determined using a two-tailed unpaired Student's *t*-test (*n* = 3, **P < 0.01, vs. saline). **(B)** Representative Western blot images (B1) and quantification (B2) of phosphorylated GCN2 (p-GCN2) levels in P1 cardiomyocytes treated with glycine (10 mM, 24 hours), the GCN2 agonist halofuginone (Halo, 20 nM, 24 hours) and PBS (control) treatment. Data are presented as mean ± SEM. Statistical significance was determined using a one-way ANOVA followed by Tukey's multiple-comparison test (*n* = 3, **P < 0.01, vs. PBS, ^##^*P* < 0.01, vs. glycine). **(C-E)** Representative immunofluorescence images (C1-E1) and corresponding quantification (C2-E2) of Ki67⁺, pH3⁺, and Aurora B⁺ cardiomyocytes in P1 cardiomyocytes treated with glycine (10 mM, 24 hours), the GCN2 agonist halofuginone (halo, 20 nM, 24 hours), or PBS. Scale bar = 20 μm (Ki67 and pH3) and 5 μm (Aurora B). Data are presented as mean ± SEM. Statistical analysis was performed using a one-way ANOVA followed by Tukey's multiple-comparison test (*n* = 5, **P < 0.01, ***P < 0.001 vs. PBS; ^###^*P* < 0.001 vs. glycine)** (F)** Representative Western blot images (A1) and quantification (A2) of phosphorylated AKT (p-AKT) levels in P1 cardiomyocytes treated with glycine (10 mM, 24 hours), the GCN2 agonist halo (20 nM, 24 hours) and PBS. Data are presented as mean ± SEM. Statistical significance was determined using a one-way ANOVA followed by Tukey's multiple-comparison test (*n* = 3, **P < 0.01, vs. PBS, ^##^*P* < 0.01, vs. glycine). **(G)** Representative immunofluorescence images (G1) and quantification (G2 and G3) of cytoplasmic co-localization of GCN2 and AKT in cardiomyocytes with glycine (10 mM) and PBS treatment. Scale bars represent 10 μm in the first and third rows, and 4 μm in the second and fourth rows. **(H)** Co-immunoprecipitation (Co-IP) analysis of GCN2 and AKT in myocardial tissue 14 days post-MI, with glycine (700 mg/kg/day, i.p.) and saline treatment.

To further elucidate how cardiomyocytes sense intracellular glycine and transmit this signal to activate the PI3K-AKT pathway, we focused on General Control Nonderepressible 2 (GCN2), a well-characterized amino acid sensor. GCN2 is activated through its binding to uncharged transfer RNAs (tRNAs) that accumulate under conditions of amino acid deficiency, leading to a conformational change that activates its kinase domain 30. Previous studies have shown that GCN2 can detect intracellular amino acid availability and modulate AKT activity through direct protein-protein interaction 31, 32. We hypothesized that GCN2 functions as a glycine sensor in cardiomyocytes, thereby coupling intracellular amino acid sensing to AKT pathway activation. Our further study found that the phosphorylation status of GCN2 in myocardial tissue was decreased following glycine supplementation (Figure 6A). To investigate whether the pro-proliferative effect of glycine is mediated through the amino acid sensor GCN2, we pharmacologically activated GCN2 using halofuginone (20 nM) 33, 34, and confirmed activation by Western blot analysis (Figure 6B). GCN2 activation abolished the proliferative effect of glycine, as evidenced by a significant reduction in the proportions of Ki67^+^, pH3^+^ and Aurora B^ +^ cardiomyocytes (Figure 6C-E). Importantly, pharmacological activation of GCN2 also blocked glycine-induced AKT phosphorylation (Figure 6F), further establishing a functional link between amino acid sensor GCN2 and AKT signaling in cardiomyocytes. To further explore the interplay between GCN2 and AKT in cardiomyocytes, we performed immunofluorescence. The results showed strong cytoplasmic co-localization of the two proteins, indicating their potential molecular interaction in cardiomyocytes. Importantly, glycine treatment markedly reduced this co-localization (Figure 6G), suggesting that elevated glycine disrupts the GCN2-AKT association and facilitates AKT activation. Critically, this physical association was further confirmed by co-immunoprecipitation (Co-IP) analysis in myocardial tissue, which demonstrated a direct GCN2-AKT interaction *in vivo* (Figure 6H).

In conclusion, these results suggest that GCN2 acts as a critical intracellular sensor of glycine in cardiomyocytes and mediates downstream AKT activation through direct interaction, thereby contributing to the pro-proliferative and regenerative effects of glycine in the heart.

### LNP@Gly delivery system improves therapeutic efficiency

**Figure 7:**
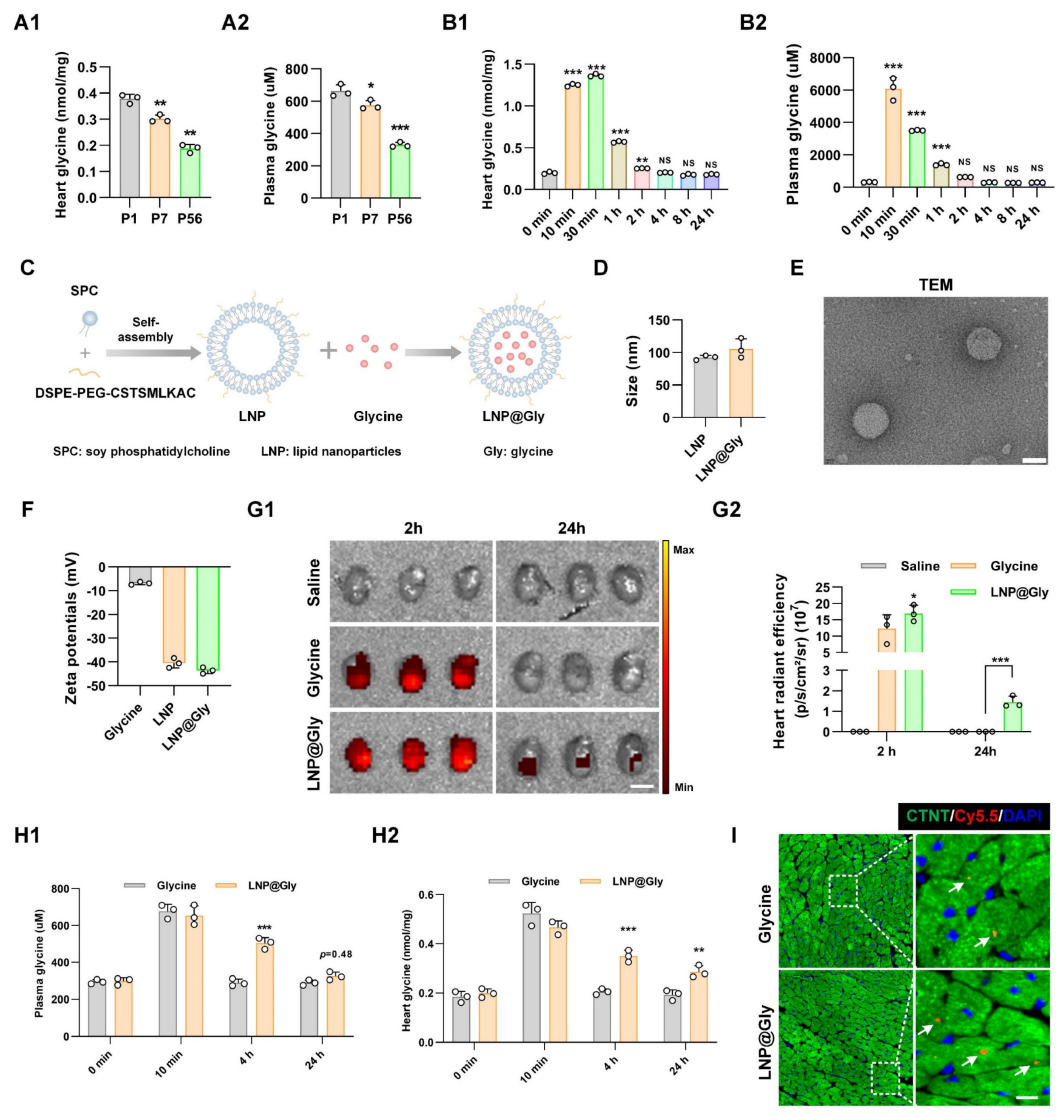
** Characterization and biodistribution of the LNP@Gly delivery system. (A)** Quantification of glycine levels in cardiac tissue (A1) and serum (A2) from P1, P7, and P56 mouse hearts. P1, P7, and P56, postnatal day 1, 7, and 56. Data are presented as mean ± SEM. Statistical analysis was performed using one-way ANOVA followed by Tukey's multiple-comparison test (*n* = 3, *P < 0.05, **P < 0.01, ***P < 0.001 vs. P1, NS = Not significant vs. P1). **(B)** Time-course analysis of glycine levels in cardiac tissue (B1) and serum (B2) following exogenous glycine supplementation (700 mg/kg, i.p.) in adult mice. Data are presented as mean ± SEM. Statistical analysis was performed using one-way ANOVA followed by Tukey's multiple-comparison test (*n* = 3, **P < 0.005, ***P < 0.001 vs. 0 min, NS = Not significant vs. 0 min). **(C)** Schematic illustration of the preparation procedure for cardiac-targeted liposomal nanoparticles encapsulating glycine (LNP@Gly). **(D)** Dynamic light scattering (DLS) analysis of the hydrodynamic diameter of LNP and LNP@Gly nanoparticles. **(E)** Representative transmission electron microscopy (TEM) image of LNP@Gly nanoparticles. Scale bar = 5 nm. **(F)** Zeta potential measurement of LNP and LNP@Gly nanoparticles. **(G)** *Ex vivo* fluorescence imaging (G1) and quantification (G2) at 2 and 24 hours after intravenous administration of Cy5.5-labeled free glycine, LNP@Gly (equivalent glycine dosage of 4 mg/kg, i.v.) or saline (control). Color scale, Min = 5.0 × 10^8^, Max = 1.3× 10^9^. Data are shown as mean ± SEM. Differences among groups were analyzed by two-way ANOVA with Sidak's post-hoc multiple-comparison test (*n* = 3, *P < 0.05, ***P < 0.001 vs. glycine). **(H)** Quantitative analysis of glycine concentrations in serum (H1) and cardiac tissue (H2) at different time points after intravenous administration of free glycine or LNP@Gly (equivalent glycine dosage of 4 mg/kg, i.v.). Data are shown as mean ± SEM. Differences among groups were analyzed by two-way ANOVA with Sidak's post-hoc multiple-comparison test (*n* = 3, *P < 0.05, ***P < 0.001 vs. glycine). **(I)** Representative immunofluorescence images of intracellular localization of Cy5.5-labeled glycine within cardiomyocytes. Scale bar = 5 µm. The white arrows indicate the intracellular Cy5.5-labeled glycine.

In line with the metabolomics data^17^, ^18^, we observed a postnatal decrease in glycine levels in both plasma and heart tissue (Figure 7A). Glycine supplementation effectively promoted cardiomyocyte proliferation. However, the short half-time of glycine limits its use in clinic. Pharmacokinetic studies showed that serum glycine peaked sharply (~6000 µM) within10 minutes post-injection and returned to baseline (~300 µM) within 2-4 hours. Similarly, cardiac glycine levels reached a maximum (~1.3 nmol/mg) at 30 minutes post-injection but also declined to baseline (~0.2 nmol/mg) within a few hours (Figure 7B). Such a high systemic peak concentration poses the risk of transient hyperglycinemia, whereas rapid clearance restricts sustained therapeutic efficacy. To overcome these limitations, we engineered a cardiac-targeted glycine delivery platform (LNP@Gly) based on liposomal nanoparticles surface-functionalized with a cardiac-homing peptide (CSTSMLKAC) (Figure 7C). This platform was designed to achieve sustained-release kinetics, enhance heart-specific accumulation, and reduce the therapeutic dosage requirement for glycine. The successful synthesis of CSTSMLKAC peptide and its subsequent PEGylation were confirmed by ^1^H-NMR spectroscopy (Figure S4). The hydrodynamic diameter of blank lipid nanoparticles (LNP), as determined by dynamic light scattering (DLS) analysis, was 92.11 ± 3.83 nm. Following glycine encapsulation, the diameter increased to 105.63 ± 13.38 nm (Figure 7D).

Morphological characterization through transmission electron microscopy (TEM) revealed monodisperse nanoparticles with well-defined spherical architecture (Figure 7E). Moreover, the zeta potentials of LNP and LNP@glycine were measured to be -40.48 ± 1.98 mV, and -43.6 ± 1.35mV, respectively (Figure 7F). The encapsulation efficiency and drug loading content were ultimately optimized to 64.41% and 6.44%, respectively. We investigate the stability of the LNP@Gly in saline and plasma, and found that the nano-platform remain relative stable in saline and plasma within 11 days (Figure S5) These results indicate that the successful preparation of the LNP@glycine nano-platform with favorable colloidal stability, supporting its subsequent application in evaluating cardiac effects. To further evaluate the *in vivo* safety profile of the formulation, we assessed systemic toxicity following treatment. Body weight changes and serum levels of TNF-α, IL-6, IL-1β, CRE, BUN, ALT, and AST in mice from both the LNP and LNP@Gly groups were comparable to those in the saline group. Moreover, histopathological examination revealed no detectable tissue damage in major organs from the LNP and LNP@Gly groups, which were similar to the saline-treated controls (Figure S6), suggesting that LNP@Gly exhibits favorable biocompatibility for *in vivo* applications.

To evaluate the targeted delivery capacity of LNP@Gly accumulation in the myocardium, intravenous administration of Cy5.5-labeled glycine and LNP@Gly (administered at an equivalent glycine dosage of 4 mg/kg) was performed following MI surgery. *Ex-vivo* fluorescence imaging demonstrated that robust accumulation and prolonged retention of LNP@Gly in the heart. The increased LNP@Gly persisting, at least, up to 24 hours post-injection (Figure 7G), which was confirmed by quantitative of glycine levels in plasma and heart tissue (Figure 7H). Additionally, the fluorescence intensity of LNP@Gly and glycine were much higher in the liver (Figure S7), indicating that the liver is the major site of glycine metabolism. Importantly, fluorescence imaging revealed clear Cy5.5 signals specifically localized within cardiomyocytes (Figure 7I).

**Figure 8:**
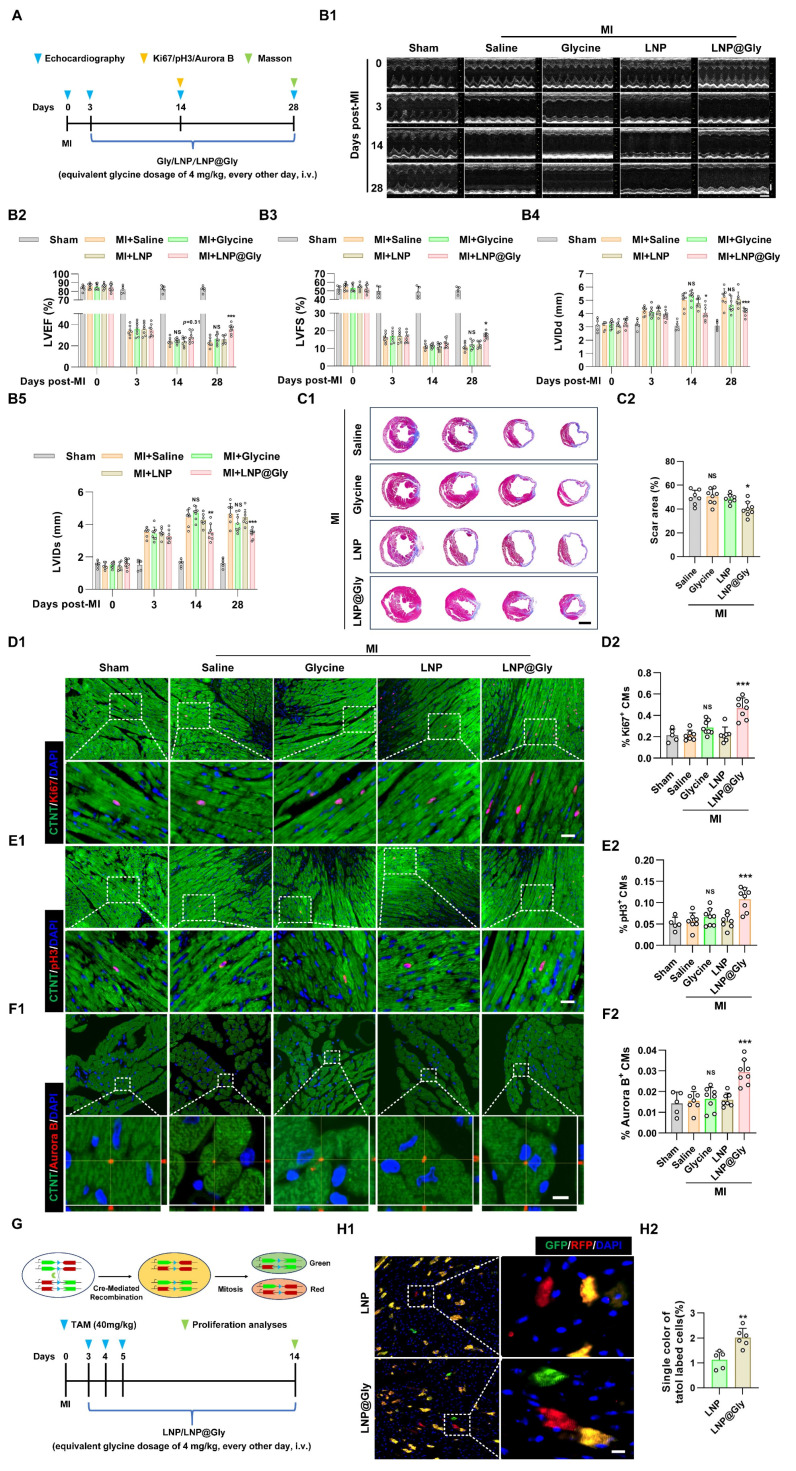
** LNP@Gly improves therapeutic efficiency at low systemic dose. (A**) Schematic diagram of the experimental design comparing free glycine and LNP@Gly treatment in the MI mouse model. **(B)** Representative echocardiographic images (B1) and quantification of left ventricular ejection fraction (LVEF %, B2), fraction shortening (LVFS %, B3), left ventricular internal diastolic diameter (LVIDd, B4) and left ventricular internal systolic diameter (LVIDs, B5) at 0, 3, 14, and 28 days post-MI, with free glycine (4 mg/kg, every other day, i.v.), LNP@Gly (equivalent glycine dosage of 4 mg/kg, every other day, i.v.) and saline (control) treatment. Data are shown as mean ± SEM. Differences among groups were analyzed by two-way ANOVA with Sidak's post-hoc multiple-comparison test (sham group, *n* = 5; MI group, *n* = 7-8, *P < 0.05, **P < 0.01, ***P < 0.001 vs. LNP, NS = Not significant vs. saline). **(C)** Representative images (C1) and quantification (C2) of Masson's trichrome staining of hearts at 28 days post-MI, with free glycine, LNP, LNP@Gly (equivalent glycine dosage of 4 mg/kg, every other day, i.v.) and saline treatment. Scale bar = 1.5 mm. Data are presented as mean ± SEM; significance was determined by one-way ANOVA followed by Tukey's multiple-comparison test (*n* = 7-8, *P < 0.05 vs. LNP, NS = Not significant vs. saline).** (D-F)** Representative immunofluorescence images (D1-F1) and corresponding quantification (D2-F2) of Ki67⁺, pH3⁺, and Aurora B⁺ cardiomyocytes in hearts 14 days post-MI treated with free glycine, LNP, LNP@Gly (equivalent glycine dose of 4 mg/kg, every other day, i.v.), or saline. Scale bar = 20 μm (Ki67 and pH3) and 5 μm (Aurora B). Data are presented as mean ± SEM; significance was determined by one-way ANOVA followed by Tukey's multiple-comparison test (sham group, *n* = 5; MI group, *n* = 7-8, ***P < 0.001 vs. LNP, NS = Not significant vs. saline). **(G)** Schematic illustration of the MADM mouse model and experimental workflow used to trace cardiomyocyte proliferation. **(H)** Representative images (H1) and quantification (H2) of Mosaic Analysis with Double Markers (MADM) lineage-tracing mouse hearts at 14 days post-MI, with LNP and LNP@Gly treatment. Scale bar = 10 μm. Data are presented as mean ± SEM. Statistical significance was determined using a two-tailed unpaired Student's *t*-test (*n* = 5-6, **P < 0.01).

To explore whether the LNP@Gly enhances therapeutic efficacy, both free glycine and LNP@Gly were administered via tail vein injection at equivalent low glycine doses (4 mg/kg), 175-fold lower than that used in Figure 3 (700 mg/kg, i.p.), every other day following MI (Figure 8A). In contrast to free glycine at lower glycine dose treatment, which had no effect on cardiac function and cardiomyocyte regeneration, LNP@Gly showed improved left ventricular systolic function (Figure 8B), and reduced infarct size (Figure 8C), accompanied with increased Ki67^+^, pH3^+^ and Aurora B ^+^ cardiomyocytes (Figure 8D-F). To further validate these findings, we employed Mosaic Analysis with Double Markers (MADM) mice, in which cardiomyocytes that undergo true mitotic division are irreversibly labeled with a single fluorescent color (green or red)^35^, ^36^ (Figure 8G). Consistent with the immunofluorescence results, treatment with LNP@Gly markedly increased the proportion of singly labeled cardiomyocytes (Figure 8H), providing definitive evidence that glycine delivered via the nanoparticle system induces bona fide cardiomyocyte cell division *in vivo*.

## Discussion

Glycine is the simplest amino acid in nature, consisting of an amino group, a carboxyl group, and two hydrogen atoms bound to a single carbon atom 37. It is classified as a conditionally essential amino acid, as endogenous synthesis is insufficient to meet physiological requirements under specific pathological 38-40. Meléndez-Hevia estimated that adults require approximately 15 g of glycine daily to support collagen synthesis and other metabolic functions, yet endogenous production provides only ~3 g/day, result in a substantial gap between demand and supply 39. There is currently no convincing evidence that healthy individuals consuming a balanced diet typically develop overt glycine deficiency. However, glycine insufficiency has been documented under specific pathological or stress-related conditions. For instance, glycine depletion has been linked to impaired glutathione synthesis and oxidative stress in metabolic disorders such as diabetes and obesity 41-44. Clinical and preclinical studies further suggest that dietary glycine supplementation exerts therapeutic benefits in several disease settings, including diabetes, metabolic syndrome and nonalcoholic fatty liver disease. Specifically, glycine supplementation improves glucose homeostasis and insulin sensitivity in type 2 diabetes 45, attenuates hepatic injury and fibrosis in nonalcoholic fatty liver disease 46, 47, reduces blood pressure in patients with metabolic syndrome 48, and protects against ischemia-reperfusion injury in the heart through anti-inflammatory and anti-oxidant effects 49. Building on this evidence, our study demonstrates a previously unrecognized role of glycine in promoting cardiomyocyte proliferation and enhancing cardiac repair following myocardial infarction.

Amino acids act not only as protein building blocks but also as metabolic and signaling regulators of cell fate, energy homeostasis, redox balance, gene expression, and cell proliferation 50-52. Glycine regulates proliferation in diverse cell types, particularly in cancers such as breast, prostate, and colorectal cancers 53-55. Mechanistically, glycine supports tumor cell proliferation by fueling de novo purine biosynthesis, which sustains DNA and RNA synthesis 56, 57, promoting glutathione production to maintain redox homeostasis and protect proliferating cells from oxidative stress 58, 59, and contributing one-carbon units for epigenetic methylation 52, 60. In skeletal muscle satellite cells, glycine enhances proliferation and regenerative capacity by activating the mTORC1 signaling pathway 61. However, glycine's effect on proliferation is context-dependent, as it can also inhibit cell division in lymphocytes and salivary-gland-derived progenitor (mSGP) cells 62, 63. In the present study, we found that glycine promotes cardiomyocyte proliferation and enhances cardiac repair following myocardial infarction.

Amino acid sensing in mammalian cells is primarily mediated by two canonical intracellular pathways, the mechanistic target of rapamycin complex 1 (mTORC1) and the general control nonderepressible 2 (GCN2) kinase 64. While mTORC1 directly senses specific amino acids such as leucine, arginine, and glutamine through dedicated upstream sensors, no receptor or signaling molecule has yet been identified as a glycine-specific sensor 65. GCN2 functions as a general amino acid sensor, and is activated when uncharged tRNAs accumulate under conditions of amino acid insufficiency 31, 66. This broad sensitivity positions GCN2 as a central node for monitoring intracellular amino acid availability. GCN2-mediated amino acid sensing has been extensively studied in diverse biological contexts, including immune tolerance in T lymphocytes under tryptophan depletion 67, cytoprotective responses in arginine-deprived intestinal epithelium 68, and nutrient-responsive regulation of neuronal plasticity 69. In the heart, we found that glycine supplementation reduced GCN2 phosphorylation and weakened its association with AKT, leading to increased AKT phosphorylation and cardiomyocyte proliferation. A similar mechanism has been described in hematopoietic stem cells 31. however, unlike the Src-dependent mechanism in hematopoietic stem cells, AKT activation in cardiomyocytes proved to be PI3K-dependent, as glycine's pro-proliferative effect was completely abolished by pharmacological inhibition with LY294002. Together, these findings suggest that GCN2 acts as a conserved brake on AKT signaling, with the specific upstream mechanisms linking GCN2 relief to AKT activation being context-dependent.

Pharmacokinetic studies have consistently shown that free amino acids are rapidly absorbed, widely distributed, and quickly cleared from circulation, with plasma concentrations typically returning to baseline within hours of supplementation 70, 71. In line with these results, our present study showed that plasma glycine levels peaked approximately 10 minutes after administration and returned to baseline within 2-4 hours. Importantly, the peak concentration reached nearly 20-fold higher than basal levels, which poses the risk of transient hyperglycinemia 72. Animal studies have reported that excessive glycine supplementation can be detrimental, as supraphysiological doses are associated with growth depression, neurological disturbances, and metabolic imbalance in certain settings 73-75. Beyond the safety concern, the physiological rapid clearance of glycine also limits its therapeutic efficacy. The transient exposure window makes it difficult to sustain effective myocardial concentrations, thereby restricting its regenerative potential. To overcome these limitations, we developed a liposomal nanoparticle-based cardiac-targeted glycine delivery system (LNP@Gly). This platform confers three major advantages: (i) improved cardiac tropism through peptide-mediated targeting, ensuring higher myocardial accumulation; (ii) reduced overall dosage requirements by enhancing local bioavailability; and (iii) prolonged circulation and half-life of glycine, enabling sustained exposure at the site of injury. These features collectively allowed LNP@Gly to achieve significant therapeutic efficacy at a dose nearly two orders of magnitude lower than that required for free glycine. Notably, lipid nanoparticle (LNP) systems have already demonstrated clinical feasibility, most prominently through their use in mRNA vaccines, which highlights their safety, scalability, and translational potential 76, 77. This combining metabolic modulation with advanced nano delivery not only addresses the pharmacokinetic shortcomings of free amino acids but also paves the way toward clinically translatable strategies for enhancing cardiac regeneration.

In summary, our study demonstrates that glycine promotes cardiomyocyte proliferation and enhances cardiac repair by relieving GCN2-mediated inhibition of AKT signaling. The targeted LNP@Gly delivery system further enhances cardiac retention and achieves therapeutic efficacy at lower doses, supporting its potential as a myocardial regenerative approach. Importantly, glycine has already been evaluated in several human clinical studies (NCT03850314, NCT04443673, NCT01417481, and NCT04658134), indicating a favorable safety profile and suggesting translational potential. However, the current work was conducted exclusively in murine models, and whether glycine exerts comparable pro-proliferative or reparative effects in the human heart remains to be determined. This limitation underscores the need for future investigations in large-animal models and non-human primates, which will be critical for assessing clinical relevance and advancing glycine-based strategies toward human myocardial regeneration.

## Supplementary Material

Supplementary figures.
