# Supplementary material for: Glycine promotes cardiomyocyte proliferation and heart regeneration via the GCN2/AKT signaling axis
Source: Theranostics. 2026 Apr 23;16(11):6220–39. doi: 10.7150/thno.127992 (PMC13142237; doi:10.7150/thno.127992)
Supplement: Supplementary file 1 — Supplementary figures. [file thnov16p6220s1.pdf]

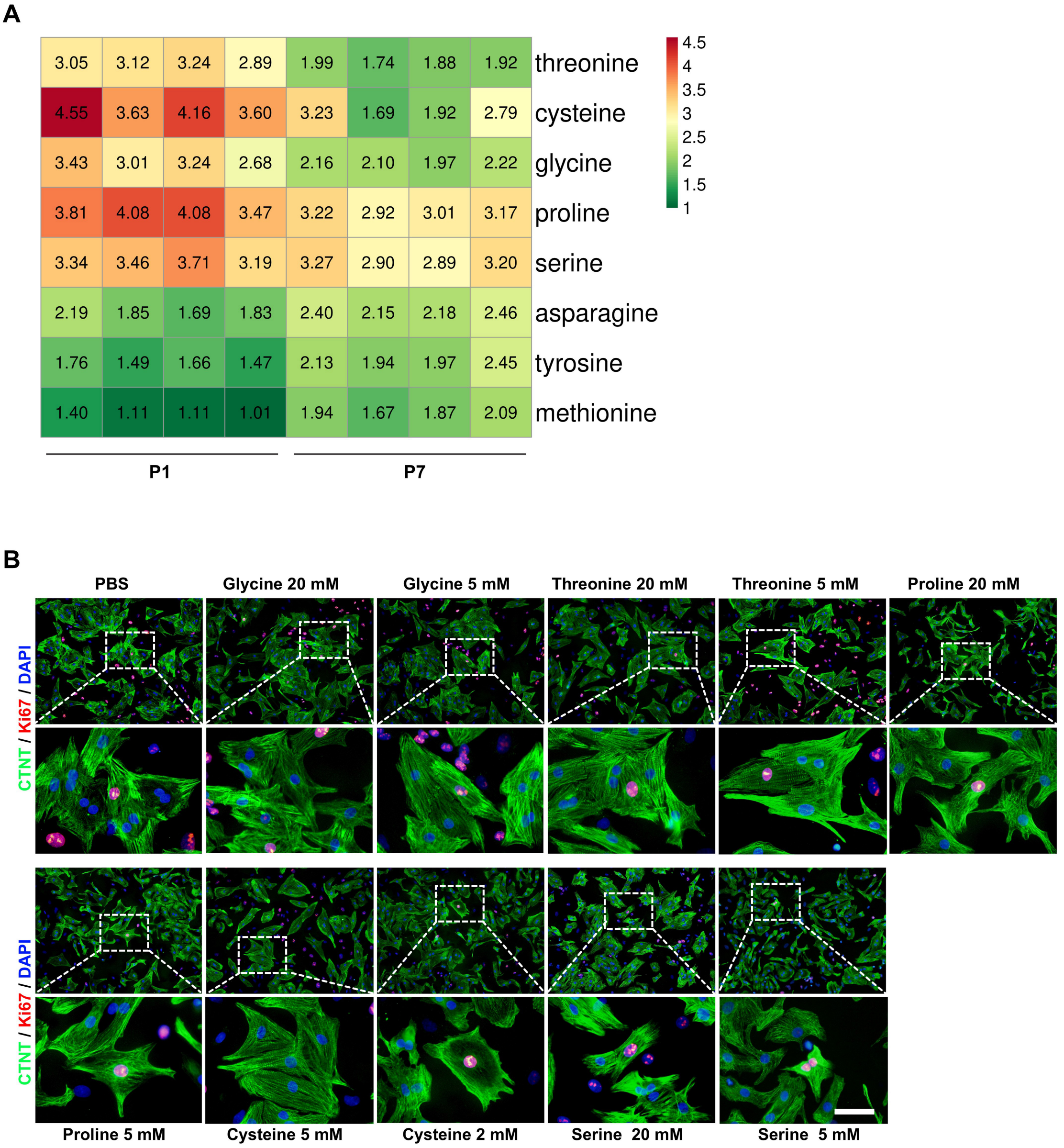

Figure S1. (A) Significantly altered amino acids in myocardial tissues from postnatal day 1 (P1) and postnatal day 7 (P7) mice. Quantitative metabolomic analysis was performed using a publicly available cardiac metabolomics dataset (Front Physiol. 2018 Apr 11;9:365), with n = 4 biological replicates per group. Values represent relative abundance. **(B)** Representative immunofluorescence images of Ki67^{+} cardiomyocytes in P7 cardiomyocytes treated with threonine, cysteine, glycine, proline, or serine (2, 5 or 20 mM) for 24 hours. Scale bar = 40 μm

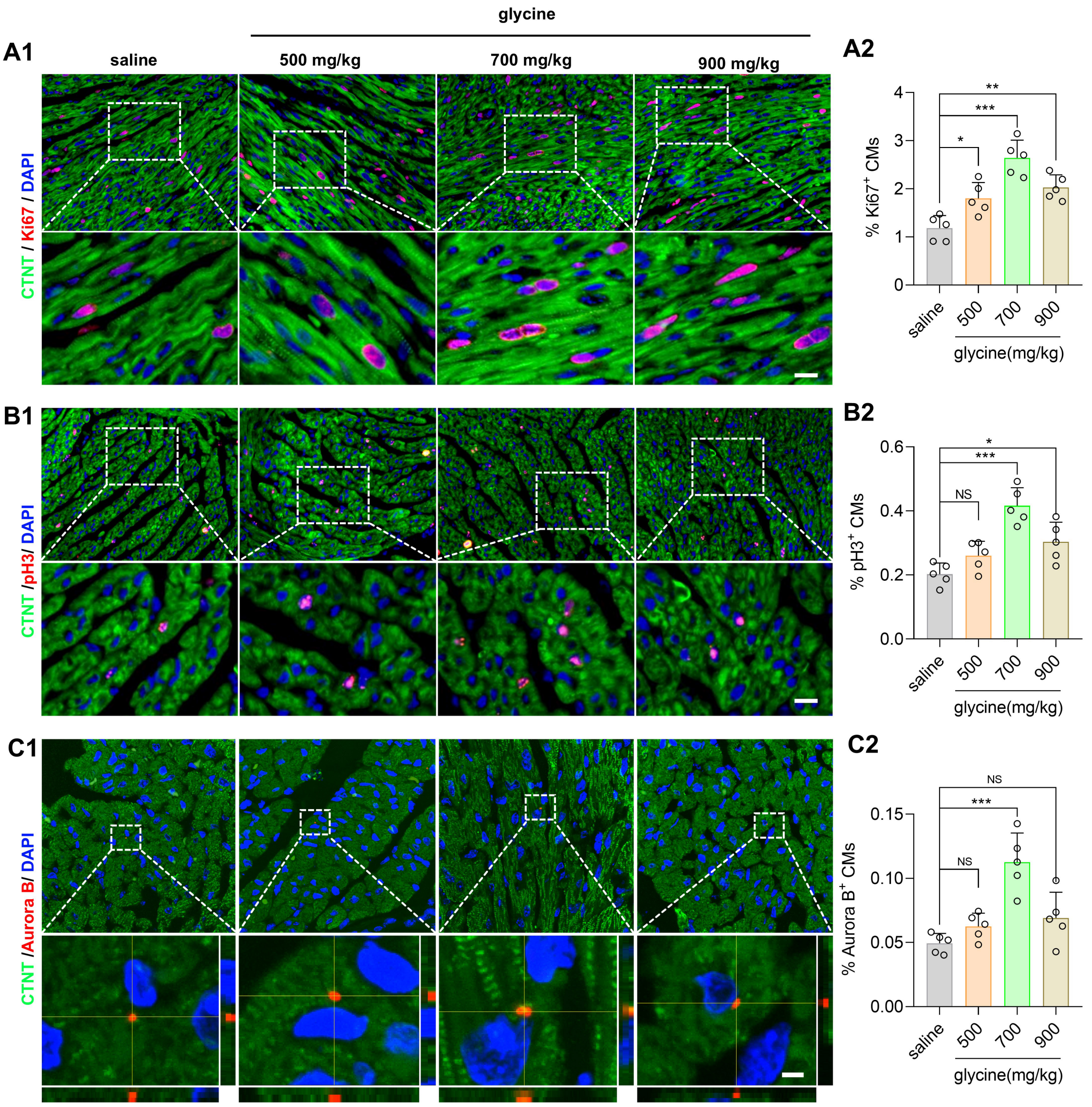

Figure S2. (A) Representative immunofluorescence images (A1) and quantification (A2) of Ki67⁺, pH3⁺, and Aurora B⁺ cardiomyocytes of P14 hearts, with different concentration of glycine (500 mg/kg, 700 mg/kg and 900 mg/kg) and saline treatment. Scale bar = 10 μm. Data are presented as mean ± SEM; significance was determined by one-way ANOVA followed by Tukey's multiple-comparison test (n = 5, *P < 0.05, **P < 0.01, ***P < 0.001).

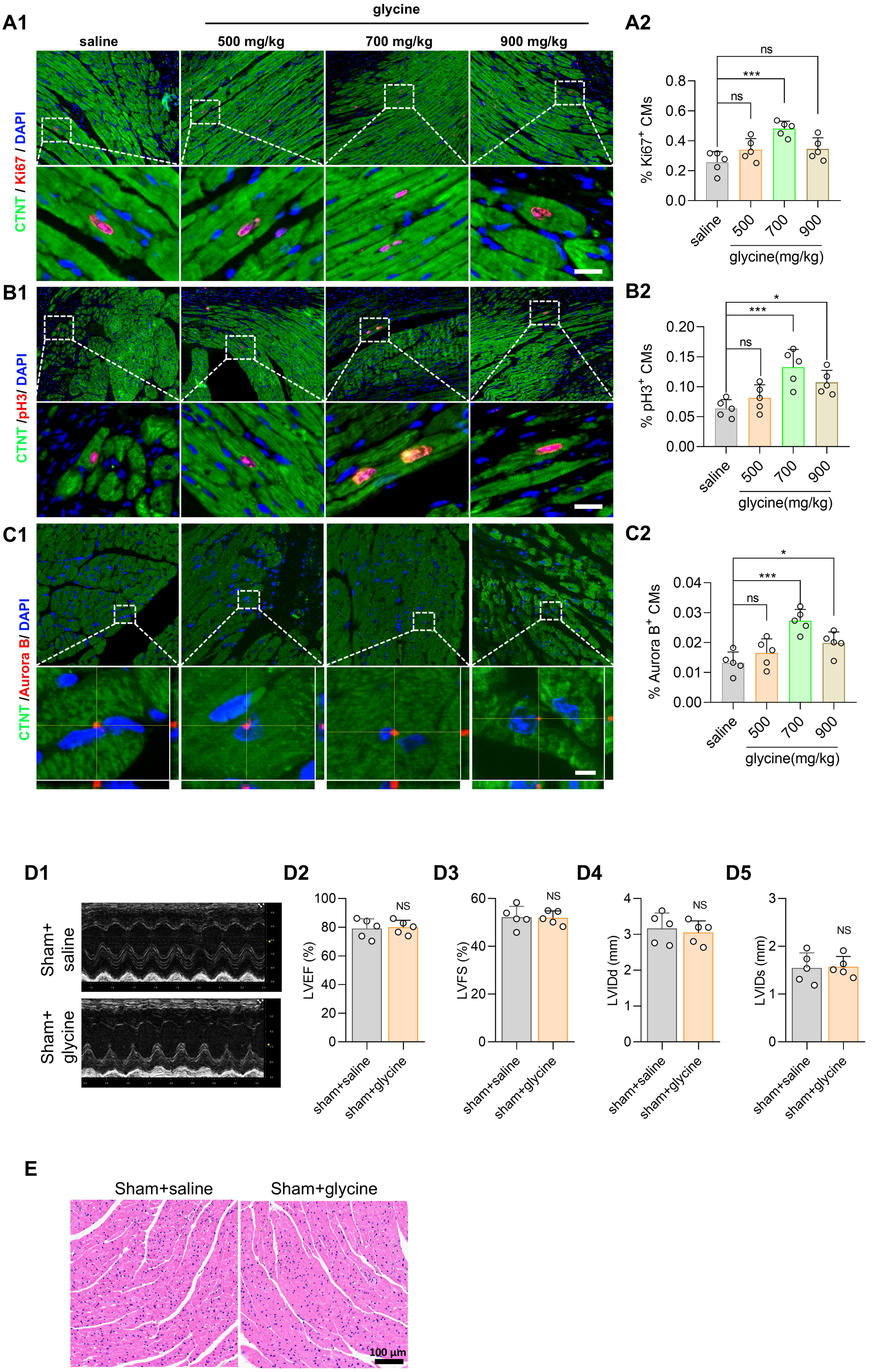

Figure S3. (A) Representative immunofluorescence images (A1) and quantification (A2) of Ki67$^+$, pH3$^+$, and Aurora B$^+$ cardiomyocytes of hearts 14

days post-MI, with different concentration of glycine (500 mg/kg, 700 mg/kg and 900 mg/kg) and saline treatment. Scale bar = 10 μm. Data are presented as mean ± SEM; significance was determined by one-way ANOVA followed by Tukey's multiple-comparison test (*n* = 5, ***P < 0.001, NS = Not significant vs. saline). **(B–C)** Representative echocardiographic images (B1) and corresponding quantitative analysis (B2-5), together with hematoxylin and eosin (H&E) staining images (C), of sham-operated mice treated with glycine (700 mg/kg) for 2 weeks, showing no detectable changes in cardiac function or myocardial structure compared with saline-treated controls. Scale bar = 100 μm.

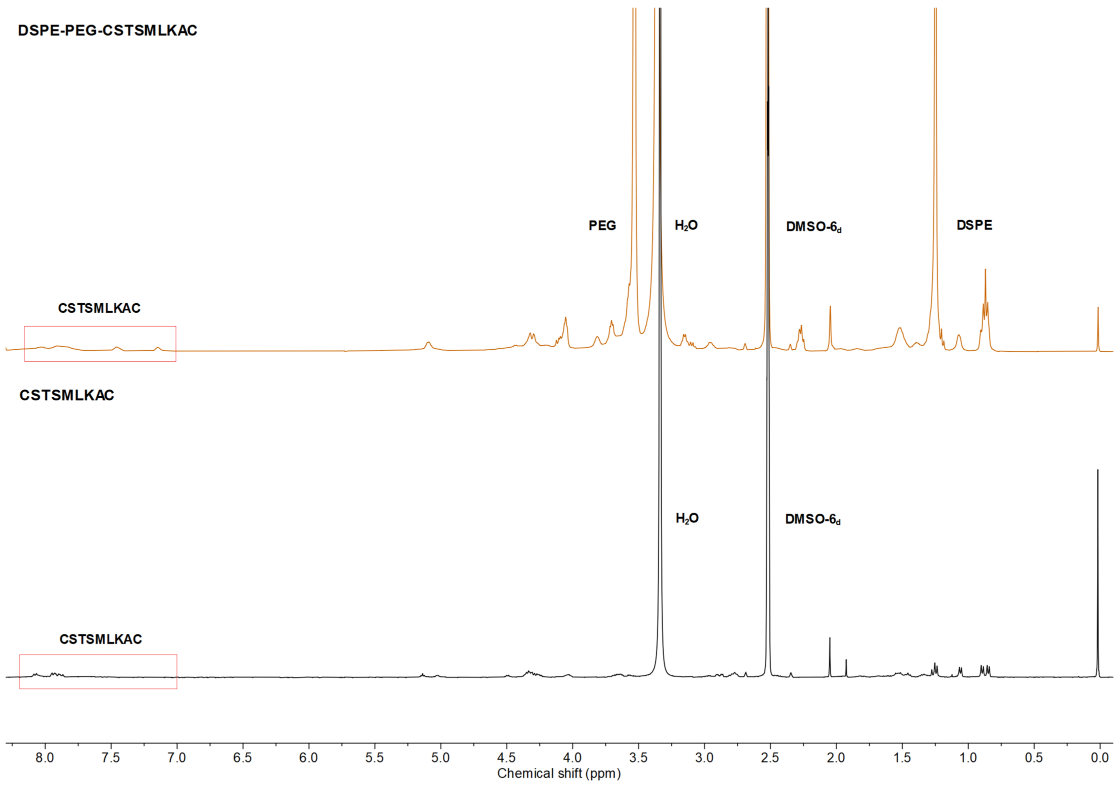

Figure S4. The 1H-NMR of the DSPE-PEG-CSTSMLKAC and CSTSMLKAC peptide.

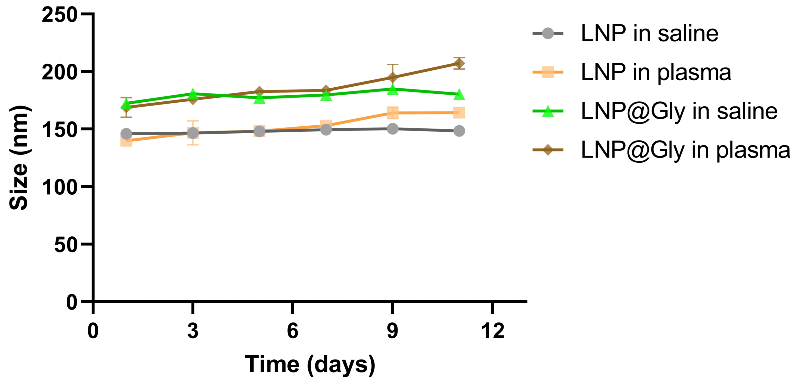

Figure S5. The stability of LNP and LNP@Gly over the course of 11 days in saline and plasma.

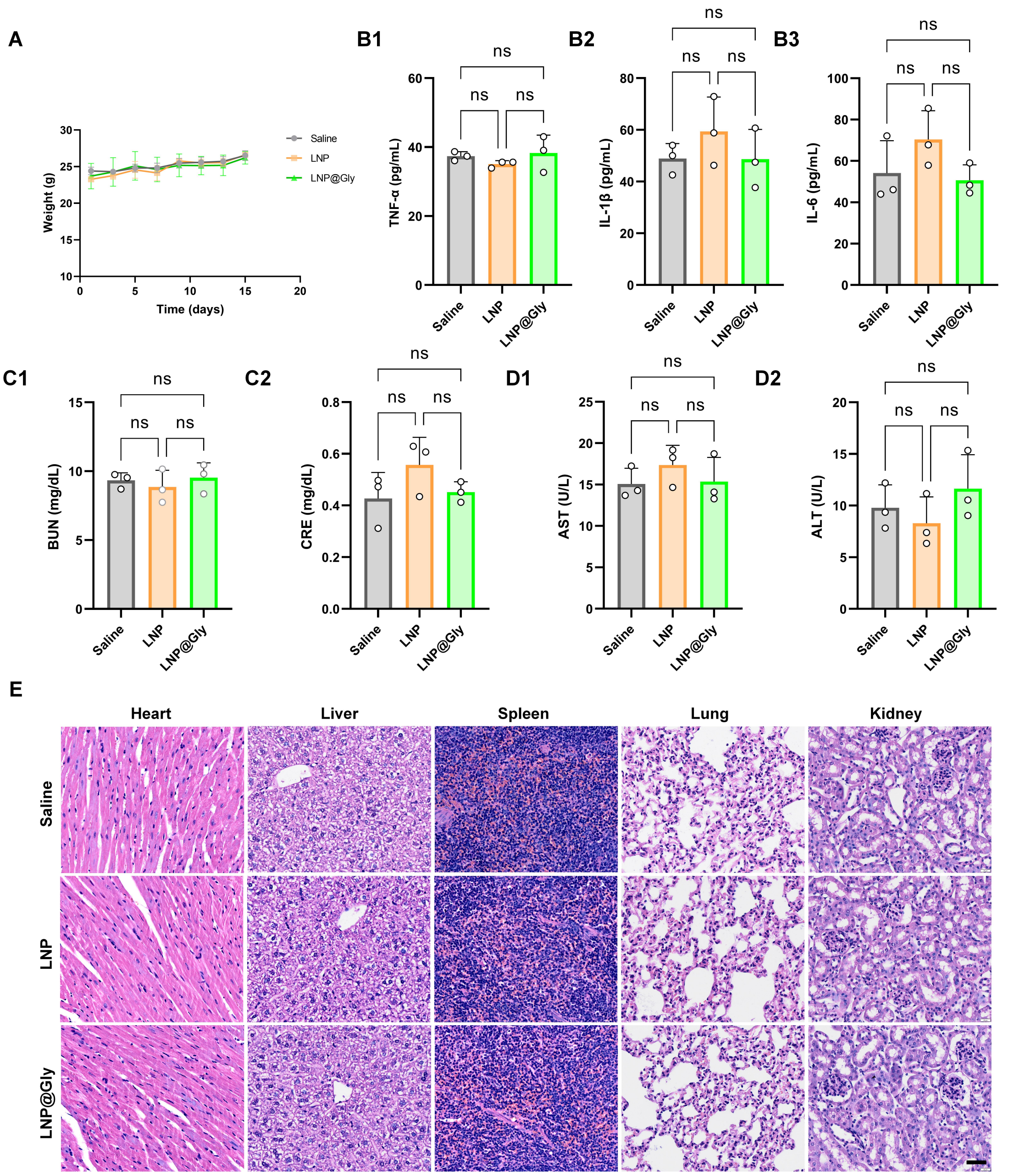

Figure S6. The biosafety evaluation of LNP@Gly. **(A)** The body weight of mice treatment with saline, LNP, or LNP@Gly for two weeks. **(B-D)** The serum levels of TNF-α, IL-6, IL-1β, CRE, BUN, ALT, and AST levels were measured of mice treatment with saline, LNP, or LNP@Gly for two weeks. Data are presented as mean ± SEM; significance was determined by one-way ANOVA followed by Tukey's multiple-comparison test (n = 3, ns, not significant). **(E)** H&E-stained histological sections from major organs (heart, liver, spleen, lung, and kidneys) of mice treatment with saline, LNP, or LNP@Gly for two weeks. Scale bar = 40 μm.

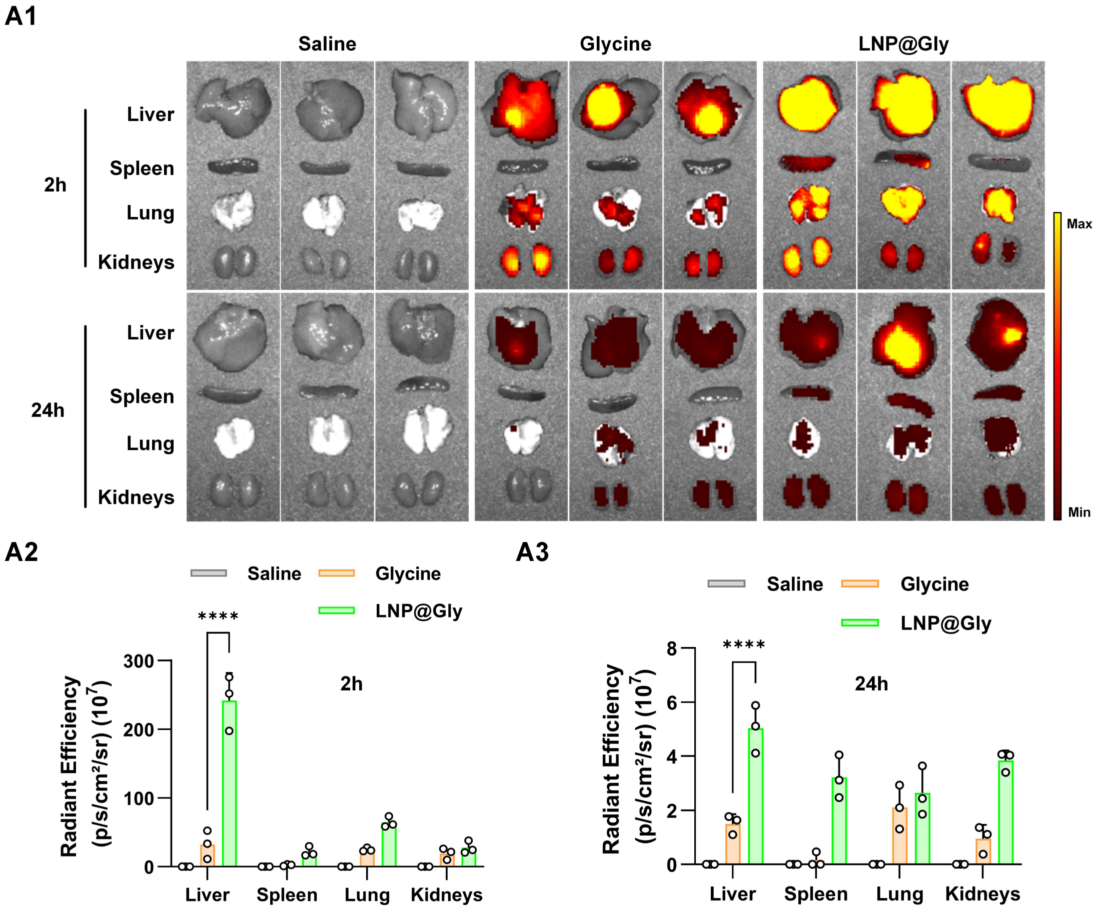

Figure S7. (A) Biodistribution and quantification of glycine-Cy5.5 and LNP@Gly-Cy5.5 that accumulated in major organs, including the liver, spleen, lung and kidneys, after 2 h and 24 h intravenous administration. Color scale, Min = 5.0×10^8, Max = 1.3×10^9. All images acquired with the same detection conditions, exposure time (t = 0.2 s), and excitation light power. Data are presented as mean ± SEM. Differences among groups were analyzed by two-way ANOVA with Sidak's post-hoc multiple-comparison test (n =3, **** $P < 0.0001$).
